# Cubic Phase‐Inducible Zwitterionic Phospholipids Improve the Functional Delivery of mRNA

**DOI:** 10.1002/advs.202413016

**Published:** 2025-02-17

**Authors:** Kazuki Iwakawa, Rikako Sato, Mariko Konaka, Yuma Yamada, Hideyoshi Harashima, Yusuke Sato

**Affiliations:** ^1^ Laboratory for Molecular Design of Pharmaceutics Faculty of Pharmaceutical Sciences Hokkaido University Kita‐12 Nishi‐6, Kita‐ku Sapporo 060‐0812 Japan; ^2^ Laboratory of Innovative Nanomedicine Faculty of Pharmaceutical Sciences Hokkaido University Kita‐12 Nishi‐6, Kita‐ku Sapporo 060‐0812 Japan

**Keywords:** cubic phase, endosomal escape, fusogenic phospholipid, lipid nanoparticles, mRNA delivery

## Abstract

Lipid nanoparticles (LNPs) are clinically advanced delivery systems for RNA. The extensively developed structure of ionizable lipids greatly contributes to the functional delivery of mRNA. However, endosomal escape is one of the severe biological barriers that continue to render this process inefficient (e.g., less than 10%). Although LNPs contain phospholipids, their role is poorly understood, and there have been few attempts to perform the chemical engineering required to improve their functionality. Herein, a cubic phase‐inducible fusogenic zwitterionic phospholipid derived from 1,2‐dioleoyl‐3‐*sn*‐glycero‐phosphoethanolamine (DOPE), DOPE‐Cx is described, that is designed to correct this problem. The orientation of a zwitterionic head group of DOPE is engineered by attaching a series of hydrophobic moieties for zwitterionic intermolecular interaction with the head structure of phosphatidylcholine (PC), and this is followed by a lipid‐phase transition into non‐lamellar phases to facilitate membrane fusion‐mediated endosomal escape. A structure–activity relationship study reveals that DOPE‐Cx lipids with small hydrophobic chains induce cubic phases instead of a hexagonal phase when mixed with PC, which enhances the functional delivery of mRNA in the liver as opposed to the action of the typically utilized and naturally occurring phospholipids. Engineered functionalized phospholipids will be of great value for the therapeutic applications of mRNAs.

## Graphical abstract

In this study, a chemically engineered zwitterionic phospholipids derived from 1,2‐dioleoyl‐3‐*sn*‐glycero‐phosphoethanolamine (DOPE), DOPE‐Cx, are designed. The DOPE‐Cx lipids induce a cubic phase upon mixing with phosphatidylcholine, facilitate membrane fusion‐mediated endosomal escape, and enhance the functional delivery of mRNA. The engineered functionalized phospholipids will be great value for the therapeutic applications of mRNAs.

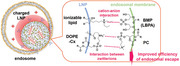

## 1 Introduction

Lipid nanoparticles (LNPs) are clinically advanced delivery systems for RNA and are expected to utilize a variety of therapeutic applications such as cancer treatment, protein replacement therapy, autoimmune disease treatment, and mRNA vaccines against infectious diseases such as COVID‐19.^[^ ^1^ ^]^ LNPs are typically composed of ionizable lipids, helper lipids, cholesterol, and polyethylene glycol (PEG) lipids. Ionizable lipids are critical components for encapsulating mRNAs via electrostatic interactions as they possess a nearly neutral charge at physiological pH (e.g., blood circulation), which improves the pharmacokinetics in the body compared with permanently charged lipids. Lipids protonated under an acidic pH, on the other hand, promote fusion with endosomal membranes wherein negatively charged lipids exist and promote a cytosolic release of mRNA for translation. Both a rational design and a combinatorial synthesis of ionizable lipids has been accomplished.^[^ ^2^ ^]^ Although the structure‐activity relationship (SAR) remains poorly understood due to the vast chemical space, a number of SAR studies are beginning to shed light on the subject.^[^ ^3^ ^]^ Also, tremendous efforts have led to significant improvements in the efficiency of functional RNA delivery and have made a critical contribution to the realization of short interfering RNA medicine (Onpattro) and mRNA vaccines (e.g., Spikevax and Comirnaty). However, the literature chronicles only a 1–3% efficiency of endosomal escape when using LNPs composed of either the commercially available ionizable lipid DLin‐MC3‐DMA or the related ionizable lipids, which suggests a dire need for significant improvement.^[^ ^4^ ^]^

In contrast to ionizable lipids, the function of helper lipids is poorly understood. Naturally occurring phospholipids are mostly utilized as helper lipids and their number is limited: 1,2‐disteroyl‐*sn*‐glycero‐3‐phosphocoline (DSPC) and 1,2‐dioleoyl‐*sn*‐glycero‐3‐phosphoethanolemine (DOPE).^[^ ^5^ ^]^ DSPC, a saturated lipid that forms a rigid membrane, contributes to the stability of LNPs. DOPE is known to be cone‐shaped due to a *cis*‐oriented unsaturated bulky hydrophobic scaffold and a hydrophilic moiety that is smaller than that of phosphocholine, which results in a fusogenic property.^[^ ^6^ ^]^ Recent work has examined the impact that unsaturation of the hydrophobic scaffold in phosphatidylcholine (PC) exerts on plasmid DNA delivery, and versions of PC with one or two unsaturated scaffolds exhibit much higher levels of transfection activity compared with that of either DSPC or DOPE both in vitro and in vivo.^[^ ^7^ ^]^ A recent study revealed the acidification‐induced structure evolution (AISE) of LNPs, which is the volume expansion and redistribution of aqueous and lipid components in response to protonation of the ionizable lipids.^[^ ^8^ ^]^ There is a strong positive correlation between the functional delivery of plasmid DNA and the degree of AISE, and this is enhanced by its fraction of unsaturated helper lipids. Thus, the role of helper lipids in LNPs has been somewhat clarified, but only to a limited extent. In addition, there have been few attempts to develop artificially functional helper lipids that provide additional value to existing LNPs.

In the above background, the identification of guidelines for the design of functional structures for artificial phospholipids was expected to provide a useful chemical space in improving the functionality of LNPs. In this study, artificial phospholipids with systematically altered structures were developed to understand the structure‐activity relationship between the induced lipid phase and the efficiency of endosomal escape.

## 2 Results and Discussion

### 2.1 Design of Novel Fusogenic Zwitterionic Lipids: DOPE‐Cx

**Figure 1:**
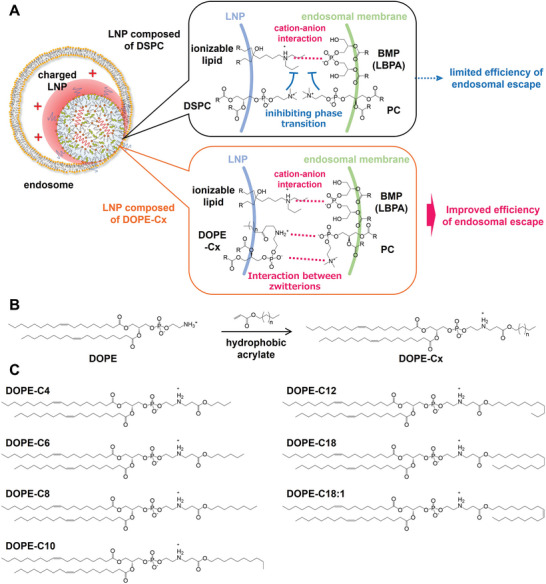
Strategy and structure of DOPE‐Cx. A) Schematic illustration of the interactive role of DOPE‐Cx. For LNPs composed of DSPC, both the DSPC in LNPs and the PC in the endosomal membrane potentially inhibit the phase transition that is driven by cation‐anion interaction. For LNPs composed of DOPE‐Cx, however, DOPE‐Cx interacts with PC in the endosomal membrane to form ion pairs, which cancels DSPC stabilization of the lamellar phase and facilitates the phase transition. B) General procedure for the synthesis of DOPE‐Cx. DOPE‐Cx lipids are synthesized via the Michell addition of a hydrophobic acrylate to DOPE. C) Chemical structure of the initial library of DOPE‐Cx.

The efficiency of functional RNA delivery has been greatly improved by the synthesis of potent ionizable lipids, because they electrostatically approach a neutral charge at physiological pH and convert to a cationic state in response to the acidification of endosomes. This triggers an electrostatic interaction between ionizable lipids and anionic lipids in endosomal membranes followed by membrane fusion‐mediated endosomal escape and the cytosolic release of payloads. However, there are prerequisites for achieving maximal efficiency. First, the pH in endosomes is sufficiently decreased in order to ionize the ionizable lipids. Second, there is a sufficient amount of anionic lipids in endosomal membranes. Third, the lipid‐phase transition to a non‐lamellar phase via the electrostatic interaction between ionizable lipids and anionic lipids is not strongly inhibited by lipids favoring the lamellar phase (i.e., phosphatidylcholine (PC)). PC is the primary phospholipid that makes up cellular membranes, and its inhibitory effect on the lipid‐phase transition in principle potentially limits the efficiency of membrane fusion strategies based on ionizable lipids. With this hypothesis in mind, we designed a series of novel zwitterionic lipids capable of interacting with versions of PC in endosomal membranes followed by an induction of the phase transition to nonlamellar phases to overcome this limitation. The zwitterionic lipids were synthesized via the one‐step Michell addition of a hydrophobic acrylate with DOPE. Strong intermolecular attractive forces are reported to exist between lipids with zwitterions that oppose one another.^[^ ^9^ ^]^ The introduction of hydrophobic groups into the primary amine of DOPE is assumed to change the orientation of phosphoethanolamine (PE) from an arrangement that is almost parallel to the dioleoyl scaffold to one that is perpendicular. The perpendicular orientation of the PE moiety facilitates the formation of an ion pair with a phosphocholine moiety of PC, which results in the formation of non‐lamellar phases. The addition of a hydrophobic group also emphasizes the cone shape of the zwitterionic lipids and contributes to the formation of non‐lamellar phases. In addition, a short carbohydrate spacer length (CSL) between the phosphate and the amine in a PE moiety is expected to facilitate ion‐pair formation with PC because shorter CSLs lower the charges in charged groups, which raises the level of hydration‐free energy and facilitates an association between zwitterions.^[^ ^10^ ^]^ The resultant zwitterionic lipids are referred to as DOPE‐Cx. **Figure** **1**A illustrates the interaction between lipids at the moment charged LNPs come into contact with endosomal membranes. Although ion pairs should form between ionizable lipids and anionic lipids in the endosomal membranes of typical LNPs composed of DSPC, the transition to non‐lamellar phases is inhibited by the large amount of endosomal PC and DSPC present in the surrounding area of LNPs. For LNPs composed of DOPE‐Cx, however, DOPE‐Cx suppresses the inhibitory effect of PC via the formation of ion‐pairs with endosomal PC, which promotes the transition to non‐lamellar phases. In the initial library of DOPE‐Cx, linear acrylates from butyl (C4) acrylate to stearyl (C18) acrylate or oleyl (C18:1) acrylate are conjugated to DOPE (Figure 1B). With the noted exception of DOPE‐C18, which is almost insoluble in alcohol, six types of DOPE‐Cx were used in subsequent experiments.

### 2.2 DOPE‐Cx Improves the Functional Delivery of mRNA to the Liver

**Figure 2:**
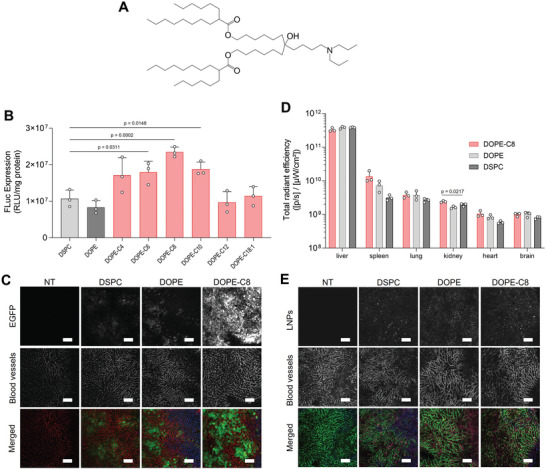
Impact that the chemical structure of DOPE‐Cx exerts on the hepatic delivery of mRNA. A) Chemical structure of ionizable lipid CL4F6 used in this study. B) Fluc expression in liver 6 h after an intravenous injection of Fluc mRNA‐loaded LNPs composed of each indicated phospholipid. Dunnett's test versus DSPC; *n* = 3. C) Observation of EGFP expression in the liver 24 h after an intravenous injection of EGFP mRNA‐loaded LNPs composed of each indicated phospholipid. Representative images show the EGFP‐positive areas (green), blood vessels visualized by DyLight649‐tomato lectin (red), and nuclei visualized by Hoechst33342 (blue); Scale bars: 100 µm. D) IVIS was used to measure the biodistribution of the DiR‐labeled LNPs composed of each indicated phospholipid 1 h after intravenous injection. Dunnett's test versus DSPC; *n* = 3. E) CLSM was used to observe the intrahepatic distribution of the DiI‐labeled LNPs composed of each indicated phospholipid 1 h after intravenous injection. Representative images show LNPs (red), blood vessels visualized by DyLight649‐tomato lectin (green), and nuclei highlighted via the use of Hoechst33342 (blue); Scale bars: 100 µm.

The DOPE‐Cx library was screened for functional mRNA delivery to the liver by LNPs in vivo. Both DSPC and DOPE were used as controls. In this study, LNPs composed of CL4F6 (**Figure** **2**A), a phospholipid, chol, and 1,2‐dimirystoyl‐*rac*‐glycero, methoxypolyethyleneglycol 2000 (PEG‐DMG) were examined at fixed molar ratios of 50, 10, 40, and 1.0, respectively. First, the firefly luciferase (Fluc) expression in liver was examined (Figure 2B). No enhancement of the expression level was observed by the replacement of DSPC with DOPE. On the other hand, the use of three DOPE‐Cx derivatives, COPE‐C6, C8, and C10, showed significantly higher expression levels compared with that when using DSPC. Both shorter (C4) and longer (C12 and C18:1) derivatives failed to enhance expression levels, which indicates a suitable range for carbon‐chain length. The DOPE‐C8, which achieved the highest expression level, was used in subsequent experiments. Next, enhanced green fluorescent protein (EGFP) expression in liver tissue was observed to verify mRNA sequence independency in the enhancement of gene expression levels by DOPE‐Cx and to identify gene‐expressing cell types (Figure 2C). All LNPs induced observable levels of EGFP expression, but DOPE‐C8‐LNPs clearly induced the highest level of expression, which was consistent with Fluc expression. When the results of both Fluc and EGFP experiments are considered, it is apparent that enhancement of mRNA expression by DOPE‐C8 is not a phenomenon that is independent of specific mRNA lengths and sequences. These results also revealed hepatocytes to be the main source of mRNA expression.

The chemical structure of phospholipids is known to affect the biodistribution of LNPs via alteration of the biomolecular corona.^[^ ^5^ ^]^ Next, we examined the effect of DOPE‐C8 on the biodistribution of LNPs. The blood half‐life of the DOPE‐C8‐LNPs was clearly less than 10 min (Figure S1, Supporting Information); therefore, the biodistribution of the LNPs 1 h after intravenous injection was examined using IVIS (Figure 2D). The DOPE‐Cx showed a significantly higher level of accumulation in the kidney compared with that when using DSPC, albeit at lower values. No differences between LNPs were observed for other organs. The highest accumulation was in the liver, which was followed by slight accumulations in the spleen and lungs. Intrahepatic distribution of the LNPs revealed that consistent levels of all LNPs accumulate mainly in hepatocytes, which are located in an area enclosed by blood vessels (Figure 2E). These results suggest that DOPE‐C8 has a limited effect on the biodistribution of LNPs and that the improved mRNA expression level derives from intracellular processes such as endosomal escape.

When nanoparticles such as LNPs are exposed to proteins such as blood, they form a protein‐based layer on their surface, which is referred to as the biomolecular corona.^[^ ^11^ ^]^ The physicochemical properties of LNPs affect their interactions with proteins and alter the biomolecular corona. The properties of the biomolecular corona are known to alter both the pharmacokinetics and the toxicity of LNPs.^[^ ^12^ ^]^ Reports also show that phospholipids such as DSPC are preferentially located in the outer layer of LNPs due to their bulky hydrophilic moiety compared with that of other lipidic components such as ionizable lipids and cholesterol.^[^ ^13^ ^]^ Negatively charged phospholipids are known to selectively deliver mRNA to the spleen and secondary lymphoid organs.^[^ ^14^ ^]^ We recently reported that the increased surface density of electrostatically neutral DSPC selectively delivers mRNA to the spleen rather than to the liver by abolishing ApoE‐dependent hepatic clearance.^[^ ^15^ ^]^ Thus, phospholipids have a significant influence on the quality of the biomolecular corona, which has a major impact on the behavior of LNPs in the body and on functional mRNA delivery. Therefore, it could be surprising that the replacement of DSPC with DOPE‐C8 had a small effect on the biodistribution of LNPs as well as on the functional delivery of mRNA. Although experimental validation is required, it is possible that DOPE‐Cx, a derivative of DOPE with a hydrophobic hydrocarbon chain attached to its hydrophilic head, alleviated the preferential localization to the LNP outer layer. Phospholipid‐free or ‐low LNPs undergo ApoE‐dependent rapid hepatic clearance.^[^ ^16^ ^]^ Therefore, in the present study LNPs containing 10 mol% DOPE‐C8 might not have shown significantly different in vivo behavior. On the other hand, the effect on the biomolecular corona in formulations with high DOPE‐Cx content or in combination with other phospholipids is interesting and merits further study.

### 2.3 Impact of DOPE‐Cx on Lipid‐Phase Transition

**Figure 3:**
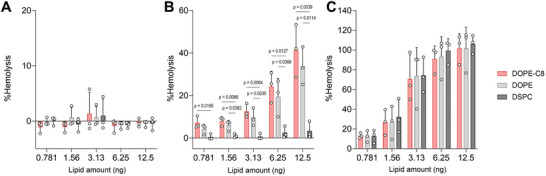
Impact of phospholipids on hemolytic activity. LNPs with the indicated amount of total lipids were mixed with a fixed amount of red blood cells at pH values of A) 7.4, B) 6.0, and C) 5.0. The percentage of hemolysis was measured after 30 min of incubation at 37 °C. Dunnett's test versus DSPC; *n* = 3.

The above results suggest that DOPE‐Cx enhances the endosomal escape of LNPs, and hemolysis assay was performed to examine any disruption of the pH‐dependent membrane activity of the LNPs in vitro (**Figure** **3**).^[^ ^17^ ^]^ No hemolysis was detected at pH 7.4, which mimics the natural pH environment in blood. This result suggests there are limits to the disruptive interaction of the LNPs with red blood cells during circulation (Figure 3A). The apparent acid dissociation constant (p*K*a) values of the tested LNPs fell within the range of 6.2–6.3, and it is reasonable to assume there was no strong interaction with the negatively charged membranes of red blood cells through electrostatic attractive force. DOPE‐Cx binds with net neutral phospholipids (e.g., PC) through zwitterionic interaction, which potentially causes a non‐specific interaction with biomembranes in a charge‐independent manner. However, it is important to note that no evidence suggests that DOPE‐C8‐LNPs facilitate hemolysis at pH 7.4, which suggests that the presence of DOPE‐C8 in LNPs is insufficient to achieve either membrane fusion or disruption without an ionizable lipid‐derived positive charge‐driven interaction. This is probably due to the relative difficulty of achieving sufficient proximity between the LNP surface where DOPE‐Cx is present and the biomembranes without electrostatic interactions acting over long distances. This is because biomolecular coronas are formed on the LNP surface, and membrane proteins and glycans are present on the biomembranes, which is the reason the zwitterionic interaction in Figure 1A only operates in the presence of a cation‐anion interaction. At pH 6.0, however, DOPE‐C8 significantly enhanced hemolytic activity compared with that using DSPC. And that level of pH mimics the pH environment in early endosomes. Some reports suggest that either rapid membrane trafficking through an organized endocytic pathway to lysosomes or rapid exocytosis and late endosome/lysosome formation are essential for the functional delivery of exogenously presented mRNA and that escape from early endosomes is important to functionally deliver RNAs into the cytosol.^[^ ^4^, ^18^ ^]^ It is reasonable to assume that endosomes become multivesicular bodies during their maturation and that the positively charged LNPs have the potential to interact with multiple luminal vesicles during the late stages of endosomes, which would potentially decrease the opportunities to deliver payloads into the cytosol. The DOPE‐LNPs also showed significant hemolytic activity at pH 6.0 despite the lack of in vivo enhancement of mRNA expression levels compared with that of DSPC‐LNPs. A primary amino moiety of PE covalently binds to complement protein C3 via nucleophilic attack on thioesters within the C3, which causes a dissociation between in vitro hemolysis assay and in vivo activity.^[^ ^19^ ^]^ All LNPs induced strong and consistent hemolytic activity at pH 5.0, which indicates that highly positive charges derived from ionizable lipids is dominant for hemolytic activity. These observations are reasonable because the molar ratio of ionizable lipids was fivefold higher than that of phospholipids in the tested LNPs and that more than 90% of the ionizable lipids took on a positively charged form at acidic pH. In other words, the DOPE‐Cx plays a supporting role in situations where ionizable lipids have a limited positive charge that prevents them from inducing either sufficient membrane fusion or disruption on their own.

**Figure 4:**
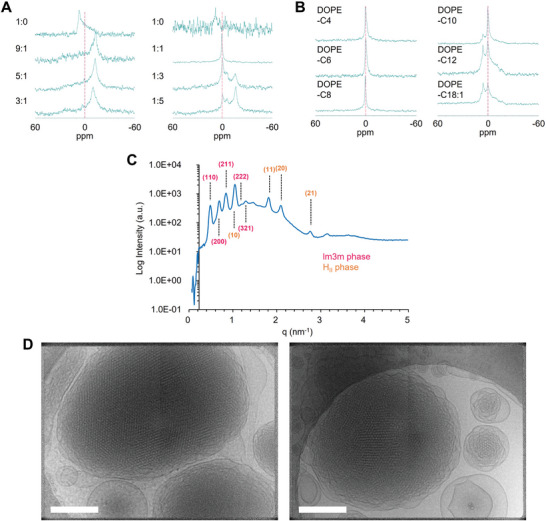
Analysis of the lipid phase of DOPE‐Cx lipid mixed with POPC. A) ^31^P NMR spectra of lipid suspensions of DOPE (left) and DOPE‐C8 (right) mixed with POPC at the indicated molar ratio. B) ^31^P NMR spectra of a lipid suspension of DOPE‐Cx mixed with POPC at a fixed molar ratio (1:1). C) SAXS profile of the DOPC‐C8/POPC (1:1) suspension. D) Cryo‐TEM observation of DOPE‐C8/POPC (1:1) nanoparticles stabilized with 0.75 mol% of PEG‐DMG. Scale bars: 200 nm.

Next, lipid polymorphism was analyzed via ^31^P NMR to understand the impact of the chemical structure of DOPE‐Cx when mixed with PC, which are the dominant electrostatically neutral phospholipids in biomembranes. In this study, POPC, which has one unsaturated fatty acid at the *sn*‐2 position, was used as a model PC. DOPE itself showed a hexagonal (H) phase characterized by a broad peak with a shoulder on the low‐field side, which then was clearly converted to a lamellar (L) phase characterized by a broad peak with a shoulder on the high‐field side when mixed with only 10 mol% of POPC, and this is consistent with observations from a previous study (**Figure** **4**A, left).^[^ ^6^ ^]^ DOPE‐C8 itself also showed a H phase, but clearly demonstrated a sharp peak at 0 ppm when mixed with POPC at an equal molar ratio (Figure 4A, right). That result proved our hypothesis that DOPE‐Cx interacts with PC and causes it to transition to a non‐lamellar phase. Since the degree of the curvature of cubic phases ranges between those of L and H phases and both DOPE‐C8 and POPC form H and L phases, respectively, this peak was considered to originate from the cubic phases that show an isotropic peak when viewed via ^31^P NMR. Although the lamellar phase‐derived peak increased depending on the POPC ratio, a sharp peak at 0 ppm remained even when mixed with a 5‐fold higher molar amount of POPC. This suggests that DOPE‐C8 retains a nonlamellar cubic (Q) phase in the presence of an excess amount of PC and is the situation during fusion with endosomal membranes. This property favors the membrane fusion‐promoting effect compared with use of the aforementioned DOPE. Next, the effect that the hydrophobic chain length of DOPE‐Cx exerts on lipid polymorphism was examined when mixed with POPC at an equal molar ratio (Figure 4B). DOPE‐Cx lipids with a shorter chain (C4 to C8) showed a sharp peak at 0 ppm, whereas H phase‐derived broad peaks gradually increased depending on the chain length (C10 to C18:1). This change in the lipid polymorphism is logical given that longer hydrophobic chain lengths increase the volume of the hydrophobic region relative to the hydrophilic head of the DOPE‐Cx and prefer to form a lipid phase with higher curvature. The ^31^P NMR shows that the peak at 0 ppm indicates phases other than the L or H phase, but identification of the specific phase is impossible. To prove that the peak derives from the Q phase, a lipid suspension containing an equimolar mixture of DOPE‐C8 and POPC was analyzed by small‐angle X‐ray scattering (SAXS) (Figure 4C). Two phases were assigned from the scattering pattern (“Relative d value” in the table). The results showed a body‐centered Q (lm3m) phase and a H phase. The lm3m phase was identified by a lattice parameter (*d* = 12.9 nm) for the unit cubic cell, which is significantly higher than both the L and H phases, and by the presence of a unique 1/√7 peak. No clear peak derived from the H phase was observed in the earlier ^31^P NMR, but it would derive from the fact that the H phase‐derived peak is broad and therefore its detection sensitivity is lower than that of the Q phase, which shows a sharp peak. Cryogenic transmission electron microscopy (cryo‐TEM) images of the consistent lipid suspensions that were stabilized using a minimum amount of PEG lipid clearly show the internally ordered structure, which suggests the presence of a Q phase (Figure 4D). The particles reportedly coexist with vesicles and attached vesicular structures.^[^ ^20^ ^]^ Considering the fact that SAXS spectra are sensitive mainly to large particles,^[^ ^21^ ^]^ and that only small particles are measured in cryo‐TEM, the lattice parameters measured by cryo‐TEM (11.1–11.2 nm) (Figure S2, Supporting Information) and SAXS (12.9 nm) matched well. The H phase structures (the lattice parameter was 7.06 nm) were also observed partially (Figure S2, Supporting Information), consistent with the SAXS result.

Membrane fusion requires a L‐to‐inverted hexagonal (H_II_) phase transition. Intermediates in the phase transition are known to form structures referred to as interlamellar attachments (ILAs), and the ILAs have a Q phase structure.^[^ ^22^ ^]^ A recent study showed that the LNPs with a Q phase are significantly more efficacious at endosomal escape than those with traditional lipoplex structures, which is due to the fact that the cubic phase requires a low level of energy for membrane fusion and fusion‐pore formation with a target membrane.^[^ ^23^ ^]^ Indeed, nanostructures with a Q phase introduced siRNA and mRNA more efficiently than counterparts with L or H_II_ phases. Consistent results were also observed in plasmid DNA delivery.^[^ ^24^ ^]^ Also, a recent study showed that designing the lipid composition to form a Q phase at the actual intra‐endosomal pH improves mRNA transfection efficiency.^[^ ^25^ ^]^ Furthermore, induction into the inverted Q phase is known to be important for inducing membrane fusion via virus‐derived membrane fusion peptides (e.g., influenza hemagglutinin peptide and feline leukemia virus fusion peptide).^[^ ^26^ ^]^ These findings are consistent with the observation in the present study that Q phase‐exhibiting DOPE‐Cx lipids with a relatively short hydrophobic chain length enhance mRNA expression levels. As of this writing, the advantage of the Q phase in membrane fusion has been demonstrated in theoretical calculations involving either in vitro or cellular systems.^[^ ^23^, ^25^, ^27^ ^]^ Siegel first suggested that bicontinuous Q phases should be highly fusogenic.^[^ ^28^ ^]^ Zheng et al. showed that fusogenicity of LNPs can be prescribed by designing LNP nanostructures that lower the energetic cost of fusion and fusion‐pore formation with a target membrane. The inclusion of a structurally active lipid, glycerol monooleate, leads to enhanced LNP endosomal fusion, fast evasion of endosomal entrapment, and efficacious RNA delivery.^[^ ^23^ ^]^ Zhai et al. discovered a strong correlation between the mesophase transition of the LNPs during acidification and mRNA delivery. The slight molecular structural differences between the examined ionizable lipids (SM‐102 and ALC‐0315) were linked to transforming the internal structures of the LNPs from an amorphous state to the Q structures during endosomal maturation. SM‐102 LNPs showed exceptionally improved efficiency of mRNA delivery in vitro due to their ability to form a Q structure at a lower pH than that of ALC‐0315, which remained within the H structure.^[^ ^25^ ^]^ Further study revealed that cholesterol plays a crucial role in transition to the Q phase at acidic pH and in successful mRNA delivery.^[^ ^27^ ^]^ Thus, accumulating evidence shows that induction of the Q phase is important for efficient delivery of RNA, and in this context the improved efficiency of in vivo mRNA delivery established in the present study is significant. Also, MacDonald and co‐workers have vigorously investigated the relationship between lipid structure, lipid phase, and transfection activity using PC derivatives, including diC18:1‐C6PC and a combination of ethyldilauroylphosphatidylcholine (EDLPC) and ethyldioleoylphosphatidylcholine (EDOPC), which are permanently cationic and form cubic structures by mixing with the anionic lipid cardiolipin.^[^ ^29^ ^]^ Our findings are consistent with their findings on the usefulness of inducing the Q phase in nucleic acid delivery and support our understanding of the importance of the lipid phase. On the other hand, compared with their extensive work, we found that DOPE‐Cx is unique and clearly different in that it is electrostatically neutral and forms a Q structure by mixing with electrostatically neutral PCs.

### 2.4 SAR Study of the DOPE‐Cx

**Figure 5:**
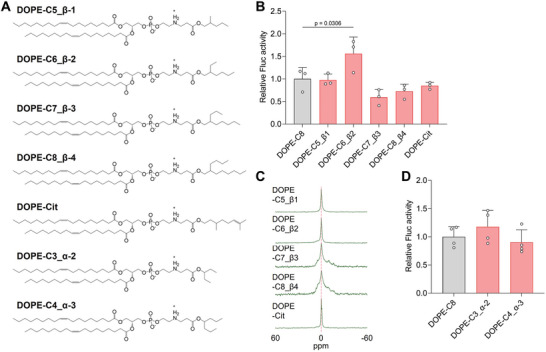
Structure‐activity relationship of DOPE‐Cx. A) Chemical structures of the additionally synthesized branched‐type DOPE‐Cx. B) Relative Fluc activity in liver 6 h after an intravenous injection of Fluc mRNA‐loaded LNPs composed of each β‐branched DOPE‐Cx; *n* = 3. C) ^31^P NMR spectra of a lipid suspension of β‐branched DOPE‐Cx mixed with POPC at a fixed molar ratio (1:1). D) Relative Fluc activity in liver 6 h following the intravenous injection of Fluc mRNA‐loaded LNPs composed of each α‐branched DOPE‐Cx; *n* = 3.

The above results revealed that the DOPE‐Cx lipids exhibiting a Q phase when mixed with POPC enhance mRNA expression in vivo. However, the structure of hydrophobic chains is limited to linear versions. To understand the SAR of branched chains, DOPE‐Cx lipids with a branched chain were additionally synthesized (**Figure** **5**A). First, four types of β‐branched lipids (C5_β‐1 to C8_β‐4) and one type of a γ‐branched lipid (Cit) were synthesized. Herein, the branched lipids will be referred to as DOPE‐C, which is followed by the “carbon number of the main chain”_“branching position”‐“carbon number of the side chain” (e.g., DOPE‐C5_β‐1). For DOPE‐Cit, the synthetic raw material citronellol (Cit) was adopted as the name. The DOPE‐C6_β‐2 showed a significantly higher mRNA expression level compared with that of DOPE‐C8, whereas the DOPE‐Cx with a longer branched chains tended to show a relatively lower expression (Figure 5B). The ^31^P NMR revealed that DOPE‐Cx with shorter β‐branched chains and DOPE‐Cit show a clear sharp peak at 0 ppm whereas those with longer β‐branched chains revealed an H phase‐derived peak, which is consistent with observations of DOPE‐Cx lipids with a linear chain (Figure 5C). Based on the mRNA expression levels for DOPE‐Cx lipids with linear and β‐branched chains, a total carbon number from 4 to 8 would be preferable. Indeed, additionally synthesized α‐branched DOPE‐Cx lipids with a total carbon number from 5 to 7 showed consistent levels of mRNA expression compared with that of DOPE‐C8 (Figure 5D). These results indicate 1) the tendency to form Q phases, 2) a relatively lower total carbon range from 4 to 8, and 3) that it is the total carbon number, i.e., bulkiness (volume occupied), rather than the hydrophobic chain length that is relevant for improved mRNA expression. This finding is consistent with the fact that the possible phases are determined by parameters such as the steric shape of the lipid, which is defined as the critical packing parameter (CPP) as proposed by Israelachvili, which was defined by the following equation: CPP = *v*/(*a*×*l*), where *v* represents the volume of the hydrophobic scaffolds, a is the cross‐sectional area of hydrophilic headgroup, and l is the length of the hydrophobic scaffolds.^[^ ^30^ ^]^

### 2.5 Characterization of the DOPE‐C8‐LNPs

**Figure 6:**
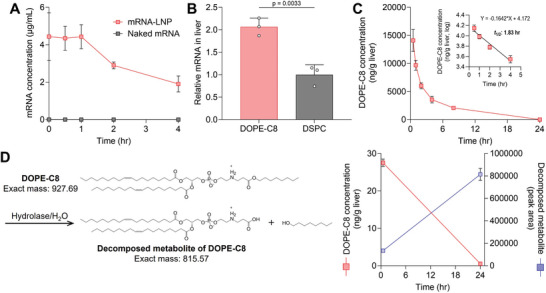
Characterization of DOPE‐C8‐LNPs. A) Stability of mRNA in freshly obtained mouse serum. Fluc mRNA‐loaded DOPE‐C8‐LNPs and naked mRNAs were separately incubated in mouse serum for the indicated time points. The concentration of the remaining mRNA was quantified via qRT‐PCR; *n* = 3. B) The relative amounts of Fluc mRNA in liver 6 h after an intravenous injection of the DOPE‐C8 or DSPC‐LNPs were compared via qRT‐PCR; *n* = 3. C) The amount of DOPE‐C8 in liver was monitored via LC/MS after an intravenous injection of the DOPE‐C8‐LNPs. The inset shows a graph of DOPE‐C8 concentrations expressed logarithmically—the half‐life was determined from the decay rate (0.5–4 h), and was highly linear; *n* = 3. D) Estimated degradation pathway of the DOPE‐C8 and structure of the decomposed metabolite (left). Quantification of both DOPE‐C8 and the metabolite after incubation of the DOPE‐C8‐LNPs in mouse serum at the indicated time points (right); *n* = 3.

Finally, DOPE‐C8‐LNPs, which were initially characterized as potent DOPE‐Cx‐LNPs, were characterized. The mRNA concentration after incubation with freshly obtained mouse serum was measured to determine the stability of the DOPE‐C8‐LNPs (**Figure** **6**A). Naked mRNA exposed to serum could hardly be detected, which indicates the instability of mRNA in biological fluid. On the other hand, mRNA encapsulated in the DOPE‐Cx‐LNPs was fully protected from enzymatic degradation for up to 2 h, which suggests the efficient encapsulation and high stability of the formulation. Next, the remaining mRNA in liver after intravenous injection was compared between DOPE‐C8‐LNPs and DSPC‐LNPs (Figure 6B). The DOPE‐C8‐LNPs showed a twofold higher level of mRNA, which indicates a more efficient cytosolic delivery of mRNA due to the fact that the hepatic accumulation level between the two LNPs was consistent. These findings suggest that the DOPE‐C8‐LNPs stably protect and deliver mRNAs to the site of action in a more efficacious manner compared with that of the original formulation.

The long‐term accumulation of ionizable lipids is known to be toxic. These compounds interact with negatively charged lipids and induce either membrane fusion or disruption. There is reasonable concern that toxicity also is induced by the long‐term accumulation of DOPE‐C8, which induces a cubic phase following interaction with PCs. Therefore, DOPE‐C8 concentrations in the liver after intravenous injection were quantified via liquid chromatography‐mass spectrometry (LC/MS) (Figure 6C). The DOPE‐C8 was rapidly eliminated and reached the lower limit of detection after 24 h. The initial (0.5–4 h) elimination half‐life was calculated to be 1.83 h. No metabolites of the DOPE‐C8 could be detected in the liver. Twenty‐four hours of incubation of DOPE‐C8‐LNPs in mouse serum resulted in a degradation of most of the DOPE‐C8 and an accumulation of the decomposed metabolites generated by hydrolytic cleavage of the hydrophobic chain, n‐octanol (Figure 6D). These results led to the conclusion that the DOPE‐C8 is susceptible to degradation by esterases present in the liver and serum, and, therefore, it is unlikely to accumulate in vivo.

Our study has some limitations. First, although a correlation was observed between the appearance of a Q phase and enhanced mRNA expression level, this result was limited in the liver and in both a fixed‐lipid composition and in the ionizable lipid structure. Comprehensive examinations of formulations and administration routes should be conducted to determine the generality inherent in the facilitating effects on functional mRNA delivery as well as to understand how to use DOPE‐Cx in order to maximize its potential functionality. Second, the fusion process of DOPE‐Cx‐containing LNPs with endosomal membranes and the detailed mechanisms involved remain unclear. Elucidating the role of the chemical structure of DOPE‐Cx in the spatiotemporal fusion process by wet experiments using model lipid membranes and in silico molecular modelling should result in ideas for the rational design of more‐functional lipids.

## 3 Conclusions

In this study, we elucidated the design of Q phase‐inducible fusogenic zwitterionic phospholipids that are referred to as DOPE‐Cx. Depending on their structures, the DOPE‐Cx lipids induced either non‐lamellar Q or H phases when mixed with net‐neutral PC and Q phase‐inducible DOPE‐Cx with smaller hydrophobic chains, which significantly enhanced the functional delivery of mRNA in the liver. Also, DOPE‐Cx was rapidly eliminated from liver tissue. These findings have important implications for the development of LNPs and should be of great value for the therapeutic applications of mRNAs using rationally engineered functionalized phospholipids.

## 4 Experimental Section

### Materials

The DOPE‐Cx series was synthesized as described in the Supporting Information. A pH‐sensitive cationic lipid, CL4F6, was synthesized as described.^[^ ^16^, ^31^ ^]^ Chol was purchased from SIGMA Aldrich (St. Louis, MO, USA). The NOF Corporation (Tokyo, Japan) supplied the following: DSPC; DOPE; POPC; and, PEG‐DMG. From Molecular Probes (Eugene, OR, USA), the following was purchased: 1,1′‐dioctadecyl‐3,3,3′,3′‐tetramethylindocarbocyanine perchlorate (DiI); 1,1′‐dioctadecyl‐3,3,3′,3′‐tetramethylindotricarbocyanine iodide (DiR); and, Ribogreen. Wako Chemicals (Osaka, Japan) supplied the 2‐(*p*‐toluidino)‐6‐napthalene sulfonic acid (TNS). Fluorescein‐labeled Lycopersicon esculentum (Tomato) lectin was purchased from Vector Laboratories (Burlingame, CA, USA). Fluc‐encoding mRNA (CleanCap, 5moU modified) and EGFP‐encoding mRNA (CleanCap, 5moU modified) were purchased from Trilink BioTechnologies. (San Diego, CA, USA). Poly(A) was purchased from SIGMA Aldrich (St. Louis, MO, USA). An iLiNP microfluidic device was fabricated and used for microfluidic mixing as described previously.^[^ ^32^ ^]^

### Preparation of RNA‐Loaded LNPs

Ethanol solutions containing CL4F6, a phospholipid, chol, and PEG‐DMG at fixed molar ratios (50:10:40:1.0) were prepared in total lipid concentrations of 8 to 16 mm. For fluorescent labeling of the LNPs, 0.5 mol% of either DiI or DiR was added to the above solutions. The RNA was dissolved in a 25 mm of acetate buffer (pH 4.0). LNPs were prepared by mixing the lipids in ethanol and RNA in an aqueous solution using a polydimethylsiloxane‐ or glass‐based iLiNP device at a total flow rate (TFR) of 0.5 or 3–5 mL min^−1^ with a flow‐rate ratio (FRR) (RNA flow rate/lipid flow rate) of 3. The nitrogen to phosphate (N/P) ratio was adjusted to 6. Syringe pumps (Harvard apparatus, MA, USA) were used to control the flow rate of the solutions. The resultant LNP solution was then dialyzed for 2 h or more at 4 °C against PBS(−) using Spectra/Por 4 dialysis membranes (molecular weight cut‐off 12000–14000 Da, Spectrum Laboratories, Rancho Dominguez, CA). The LNP solution was concentrated by ultrafiltration using an Amicon Ultra‐15 unit (MWCO 100 kDa, Millipore) as needed.

### Characterization of the LNPs

The values for ζ‐average size, polydispersity index (PdI), and ζ‐potential of the LNPs were measured using a Zetasizer Nano ZS ZEN3600 instrument (Malvern Instruments, Worchestershire, UK). The encapsulation efficiency and total concentration of mRNA were measured via Ribogreen assay, as described previously.^[^ ^33^ ^]^

In situ apparent p*K*a determination was performed by TNS assay. Briefly, 30 µm of LNP lipids and 6 ¼m of TNS were mixed in 50 µL of 20 mm citrate buffer, 20 mm sodium phosphate buffer or 20 mm Tris‐HCl buffer, containing 130 mm NaCl, at pH values ranging from 3.5 to 9.5 in a black 384‐well plate by means of epMotion 5070 (Eppendorf SE, Hamburg, Germany). Fluorescence was measured by a Varioskan Flash setup with *λ*_ex_ = 321 nm and *λ*_em_ = 447 nm at 37 °C. The p*K*a values were measured as the pH resulted in a rise to half‐maximal fluorescent intensity.

### Animals

The experimental protocols were all reviewed and approved by the Hokkaido University Animal Care Committee under the guidelines for the care and use of laboratory animals (approval number: 20‐0176). BALB/c mice (female, 4–8 weeks old) were sourced from Japan SLC (Shizuoka, Japan). Mice were maintained on a regular 12 h light/12 h dark cycle in a specific animal facility at Hokkaido University. Four to five mice were housed in each cage. The mice were fed a pelleted mouse diet (cat# 5053, LabDiet, USA) and water was available ad libitum.

### Measurement of Fluc Activity

Mice were intravenously injected with Fluc mRNA‐loaded LNPs at a dose of 0.01 mg mRNA/kg. Six hours after injection, mice were euthanized, and tissues were collected, frozen in liquid nitrogen, and stored at −80 °C. The tissues were homogenized in passive lysis buffer (Promega, Madison, WI, USA) using Micro Smash MS‐100R (TOMY Seiko Co., Ltd., Tokyo, Japan). Debris was removed by centrifugation. Fluc activity in the supernatant was measured using the Luciferase Assay System (Promega) according to the manufacturer's protocol. Luminescence was measured using a luminometer (Luminescencer‐PSN, ATTO, Japan). Protein concentrations were determined using a BCA Protein Assay Kit (Pierce, Rockford, IL, USA). Fluc activity was expressed as relative light units (RLU) per mg protein.

### Observation of Intrahepatic Distribution of LNPs

Mice were intravenously injected with the DiI‐labeled LNPs at a dose of 1.0 mg mRNA/kg. Ten minutes before the collection of liver tissues, blood vessels were stained by an intravenous injection of 40 µg of fluorescein‐labeled Tomato lectin. Liver tissues were collected 1 h after the injection of the LNPs. The liver tissues were excited with 405, 561, and 638 nm light using an LU4 4 laser unit. The intrahepatic distribution of the LNPs was observed using a Nikon A1 (Nikon Co. Ltd. Tokyo, Japan) equipped with an objective lens (PlanApo VC 20×0.75NA) and a dichroic mirror (DM 405/488/561/640). The three fluorescence detection channels (Ch) were set to the following filters: Ch1,450BP50 for Hoechst33342, Ch2,595BP50 for DiI‐labeled LNPs, Ch3,700BP75 for DyLight649‐labeled Tomato lectin.

### Observation of the Distribution of Gene Expression in the Liver

Mice were intravenously injected with the EGFP mRNA‐loaded LNPs at a dose of 1.0 mg mRNA per kg. Ten minutes before the collection of liver tissues, blood vessels were stained by an intravenous injection of 40 µg of DyLight649‐labeled Tomato lectin. Liver tissues were collected 24 h after injection of the LNPs. The liver tissues were excited with 405, 488 and 635 nm light using an LU4 4 laser unit. The intrahepatic distribution of the LNPs was observed using a Nikon A1 (Nikon Co. Ltd. Tokyo, Japan) equipped with an objective lens (PlanApo VC 20×0.75NA) and a dichroic mirror (DM 405/488/561/640). The three fluorescence detection channels (Ch) were set to the following filters: Ch1,450BP50 for Hoechst33342, Ch2,525BP50 for EGFP, Ch3,700BP75 for DyLight649‐Tomato lectin.

### Biodistribution of LNPs Using an In Vivo Imaging System (IVIS)

Mice were intravenously injected with the poly(A)‐loaded DiR‐labeled LNPs at a dose of 1.0 mg RNA per kg. Livers, spleens, brains, hearts, lungs, and kidneys were collected 1 h after the injection of the LNPs. The organs were imaged on an in vivo imaging system (IVIS Lumina III, PerkinElmer, Waltham, MA, USA).

### Hemolysis Assay

Fresh red blood cells (RBCs) were collected from BALB/c mice and suspended in buffer (10 mm malic acid, 10 mm HEPES, 10 mm MES,120 mm NaCl) at the indicated pH. The RBC suspension was mixed with each quantity of LNPs, and incubated at 37 °C for 30 min. After the incubation, the absorbance at 545 nm of the supernatant was measured after centrifugation (4 °C, 400 g, 5 min). The samples were measured following incubation with 0.5 w/v% Triton X‐100 as a positive control, and without LNPs as a negative control. The % hemolysis was represented as the % of the absorbance of the positive control.

### Determination of the Lipid Phase by ^31^P NMR Spectroscopy and SAXS

For ^31^P NMR spectroscopy, DOPE‐Cx, and POPC (16 µmol of total lipid) were dissolved in 400 µL of chloroform, and the solvent was removed on a rotovap to form lipid films. The lipid films were hydrated with 500 µL of 20 mm HEPES buffer (pH 7.4, 130 mm NaCl) at 60 °C. The lipid dispersion was transferred to NMR tubes. Proton‐decoupled ^31^P NMR spectra were obtained using an ECX 400P (JEOL) spectrometer. Acquisition parameters included 60 ° pulses, a spectral width of 280 kHz with 32768 data points, and a 1 s interpulse delay time. The temperature was regulated from 21 to 30 °C. An exponential multiplication corresponding to 50 Hz line broadening was applied to the free induction decays before Fourier transformation. The chemical shift was referenced to external 85% phosphoric acid (H_3_PO_4_).

For SAXS, a lipid suspension (32 mm lipid) composed of an equal molar ratio of DOPE‐Cx/POPC suspended in 20 mm HEPES buffer (pH7.4, 130 mm NaCl) was prepared by a procedure similar to that used for the ^31^P NMR sample. SAXS experiments were performed on a NANO‐Viewer instrument (Rigaku Corporation, Tokyo, Japan) equipped with a PILATUS detector at the Foundation for Promotion of Material Science and Technology of Japan (Tokyo, Japan). Silver behenate was used to calibrate the detector‐to‐sample distance. The sample‐detector surface distance was 1 m. Exposure time was 1 h. The intensity of scattering was integrated azimuthally on a 2D diffraction pattern and plotted as a function of the scattering vector, *q* = 4 πsin(*θ*/2)/λ (nm^−1^), with *θ* as the scattering angle. Film‐derived data were subtracted as background.

### Cryogenic Transmission Electron Microscopy (Cryo‐TEM)

Lipid suspensions composed of DOPE‐C8 and POPC (1:1 molar ratio) stabilized with 0.75 mol% of PEG‐DMG (ζ‐average: 221 nm, PdI: 0.22, measured by DLS) were added to Quantifoil Cu1.2/1.3 grids, and frozen in liquid ethane using FEI Vitrobot MarkIV. The grids were stored in liquid nitrogen until use for imaging. JEM‐2200FS (JEOL, Japan) was used to obtain the images. The instrument was operated at 200 kV. A DE‐20 camera was used to capture images. Samples were imaged at a 30000× magnification. Sample preparation and imaging was performed by Terabase Inc. (Aichi, Japan).

### Quantification of DOPE‐Cx

To measure the concentration of DOPE‐Cx in liver, mice were intravenously injected with poly(A)‐loaded LNPs at a dose of 1 mg RNA per kg. The mice were euthanized at indicated time points after the injection, and liver tissues were collected. All samples were stored at −80 °C prior to analysis for lipid quantification by LC/MS. The tissues were homogenized in 400 µL of water. The tissue homogenates (50 µL) were extracted with 400 µL of acetonitrile/isopropanol (v/v 50:50). After centrifugation (4 °C, 15000×*g*, 10 min), the supernatant was filtered, and the filtrate transferred to a vial for LC/MS analysis.

The samples were analyzed using Nexera lite (Shimazu Corporation, Kyoto, Japan) equipped with a Shim‐pack Arata C18 (2.0 × 50 mm, 5 µm) with a gradient mobile phase of water (5 mm ammonium acetate) and acetonitrile/isopropanol (v/v 1:2) (5 mm ammonium acetate) at 60 °C with a flow rate of 0.2 mL min^−1^ for 15 min. The samples were detected using an LCMS‐2050 (Shimazu Corporation) with selected ion monitoring in the positive ion mode for detection using masses of [M+H]^+^ → 928.5 (DOPE‐C8) and 816.6 (decomposed metabolite of DOPE‐C8).

### Stability Evaluation of mRNA in Serum

Mouse blood was collected from the vena cava under isoflurane anesthesia, and the serum was then isolated by centrifugation. Fluc mRNA‐loaded LNPs (167 µg mRNA mL^−1^) were added in the freshly isolated mouse serum in a volume ratio of 1:9 (LNPs:serum). The mixtures were incubated at 37 °C for the indicated time points and then stored in −80 °C. The samples were diluted 5‐fold with saline and then extracted using TRIzol LS reagent (ThermoFisher). A fixed amount of liver total RNA was extracted using TRIzol reagent (ThermoFisher) that was added to the extracted sample in order to add carrier RNAs for alcohol precipitation and to supply reference genes for qRT‐PCR. After purification of RNA by alcohol precipitation, the RNA was reverse‐transcribed to cDNA using a ReverTra Ace qPCR RT Master Mix with gDNA Remover (TOYOBO, Osaka, Japan). Quantitative PCR analysis was performed on the cDNA using the PrimeTime Gene Expression Master Mix (IDT) and the Thermal Cycler Dice Real Time System III (Takara Bio Inc., Shiga, Japan). All reactions were performed in a volume of 10 µL. The quantity of Fluc mRNA was normalized using a level of endogenous Gusb mRNA, which was expressed as the relative quantity of Fluc mRNA at each time point against the input quantity. The primers/probe for Fluc mRNA were (forward) 5′‐GCA GGA CTA CAA GAT CCA GAG‐3′, (reverse) 5′‐GGT TGC TCA GGT CGT ACT T‐3′, and (probe) 5′‐/56‐FAM/ ACC CTG TTC /ZEN/ AGC TTC TTC GCC AA /3IABkFQ/‐3′ and for murine Gusb were (forward) 5′‐ACC ACA CCC AGC CAA TAA AG‐3′, (reverse) 5′‐AGC AAT GGT ACC GGC AG‐3′, and (probe) 5′‐/56‐FAM/ ACA TCA CCC /ZEN/ AAG AAG CAG CCC T /3IABkFQ/‐3′.

### Data Analysis

Results are expressed as the mean + the SD of independent experiments. For comparisons between the means of two variables, unpaired Student's *t* tests were performed. For comparisons between multiple groups, a one‐way analysis of variance (ANOVA) followed by a Dunnett's post hoc test was performed. Group size, definition of center, and dispersion and precision measures are also noted in the figure legends as appropriate. All statistics were completed using GraphPad Prism software version 10.1.2 (San Diego, CA, USA).

## Conflict of Interest

H.H. and Y.S. are the authors of patent appl. No. JP/2023/178361 in relation to this publication.

## Author Contributions

Conceptualization and methodology: Y.S.; Validation: Y.S.; Formal analysis: K.I. and Y.S.; Investigation: K.I., R.S., M.K., and Y.S.; Resources: Y.S.; Writing‐original draft: Y. S.; Writing‐review & editing: K.I., Y.Y., H.H., and Y.S.; Visualization: Y.S.; Supervision: Y.S.; Project administration: Y.S.; Funding acquisition: H.H. and Y.S.; All authors have given approval to the final version of the manuscript.

## Supporting information



Supporting Information

## Data Availability

The data that support the findings of this study are available from the corresponding author upon reasonable request.
