# Supplementary material for: Cubic Phase‐Inducible Zwitterionic Phospholipids Improve the Functional Delivery of mRNA
Source: Adv Sci (Weinh). 2025 Feb 17;12(17):2413016. doi: 10.1002/advs.202413016 (PMC12061338; doi:10.1002/advs.202413016)
Supplement: Supplementary file 1 — Supporting Information [file ADVS-12-2413016-s001.docx]

**SUPPORTING INFORMATION**

**Cubic Phase-Inducible Zwitterionic Phospholipids Improve the Functional Delivery of mRNA**

*Kazuki Iwakawa^1^, Rikako Sato^1^, Mariko Konaka^1^, Yuma Yamada^1,2^, Hideyoshi Harashima^2^, Yusuke Sato*^1,2^*

*^1^Laboratory for Molecular Design of Pharmaceutics, Faculty of Pharmaceutical Sciences, Hokkaido University, Kita-12 Nishi-6, Kita-ku, Sapporo 060-0812, Japan*

*^2^Division of Applied Chemistry, Faculty of Engineering, Hokkaido University, Kita-13 Nishi-8, Kita-ku, Sapporo 060-8628, Japan*

*^3^Laboratory of Innovative Nanomedicine, Faculty of Pharmaceutical Sciences, Hokkaido University, Kita-12 Nishi-6, Kita-ku, Sapporo 060-0812, Japan*

**Corresponding author:*

*Yusuke Sato E-mail address: y_sato@pharm.hokudai.ac.jp Tel: +81-11-706-3734 Fax: +81-11-706-3734*

**Table S1. Physicochemical properties of the mRNA-loaded LNPs used in this study, related to Figures 2B, 2C, 5B, 5C, 6A, and 6B**

| phospholipid | ζ-Average  (nm) | Number mean  (nm) | PdI | ζ-potential  (mv) | Encapsulation  (%) | pKa |
| --- | --- | --- | --- | --- | --- | --- |
| DOPE-C4 | 87 | 58 | 0.17 | -5.01 | 91.2 | 6.30 |
| DOPE-C6 | 86 | 59 | 0.15 | -1.44 | 91.5 | 6.29 |
| DOPE-C8 | 88±11 | 61±12 | 0.13±0.06 | -6.7±3.1 | 95.2±2.8 | 6.34±0.06 |
| DOPE-C10 | 82 | 48 | 0.18 | -6.95 | 93.1 | 6.24 |
| DOPE-C12 | 100 | 59 | 0.25 | -6.31 | 94.2 | 6.29 |
| DOPE-C18:1 | 81 | 56 | 0.18 | -2.91 | 93.9 | 6.31 |
| DOPE | 90±5 | 61±6 | 0.20±0.07 | -2.9±0.3 | 94.2±1.6 | 6.38±0.07 |
| DSPC | 87±9 | 59±7 | 0.13±0.05 | -6.2±2.3 | 95.8±1.0 | 6.23±0.04 |

**Table S2. Physicochemical properties of the poly(A)-loaded LNPs used in this study, related to Figures 2D, 2E, 3, 6C, and 6D**

| phospholipid | ζ-Average  (nm) | Number mean  (nm) | PdI | ζ-potential  (mv) | Encapsulation  (%) |
| --- | --- | --- | --- | --- | --- |
| DOPE-C8 | 89±12 | 60±10 | 0.13±0.02 | -4.7±1.4 | 96.6±1.8 |
| DOPE | 82±9 | 54±4 | 0.13±0.06 | -3.1±0.8 | 98.4±1.4 |
| DSPC | 80±5 | 62±5 | 0.06±0.02 | -3.4±1.2 | 97.0±1.4 |

**Table S3. Physicochemical properties of the mRNA-loaded LNPs composed of branched DOPE-Cx used in this study, related to Figures 5B and 5C**

| phospholipid | ζ-Average  (nm) | Number mean  (nm) | PdI | ζ-potential  (mv) | Encapsulation  (%) | pKa |
| --- | --- | --- | --- | --- | --- | --- |
| DOPE-C5_β-1 | 94 | 60 | 0.21 | -6.4 | 95.9 | 6.51 |
| DOPE-C6_β-2 | 94 | 73 | 0.07 | -5.0 | 95.7 | 6.47 |
| DOPE-C7_β-3 | 79 | 61 | 0.08 | -9.6 | 96.0 | 6.48 |
| DOPE-C8_β-4 | 112 | 71 | 0.21 | -5.8 | 96.3 | 6.47 |
| DOPE-Cit | 85 | 65 | 0.07 | -6.1 | 95.9 | 6.56 |
| DOPE-C3_α-2 | 91 | 54 | 0.20 | -10.5 | 97.2 | 6.30 |
| DOPE-C4_α-3 | 91 | 52 | 0.21 | -12.0 | 97.1 | 6.30 |

**Figure S1. Time-dependent blood concentration of the DOPE-C8-LNPs.** Mice were intravenously injected with DiR-labeled DOPE-C8-LNPs at a dose of 1 mg RNA/kg. Blood was collected from the tail vein at the indicated time points, and the LNP concentration was measured; n=3.


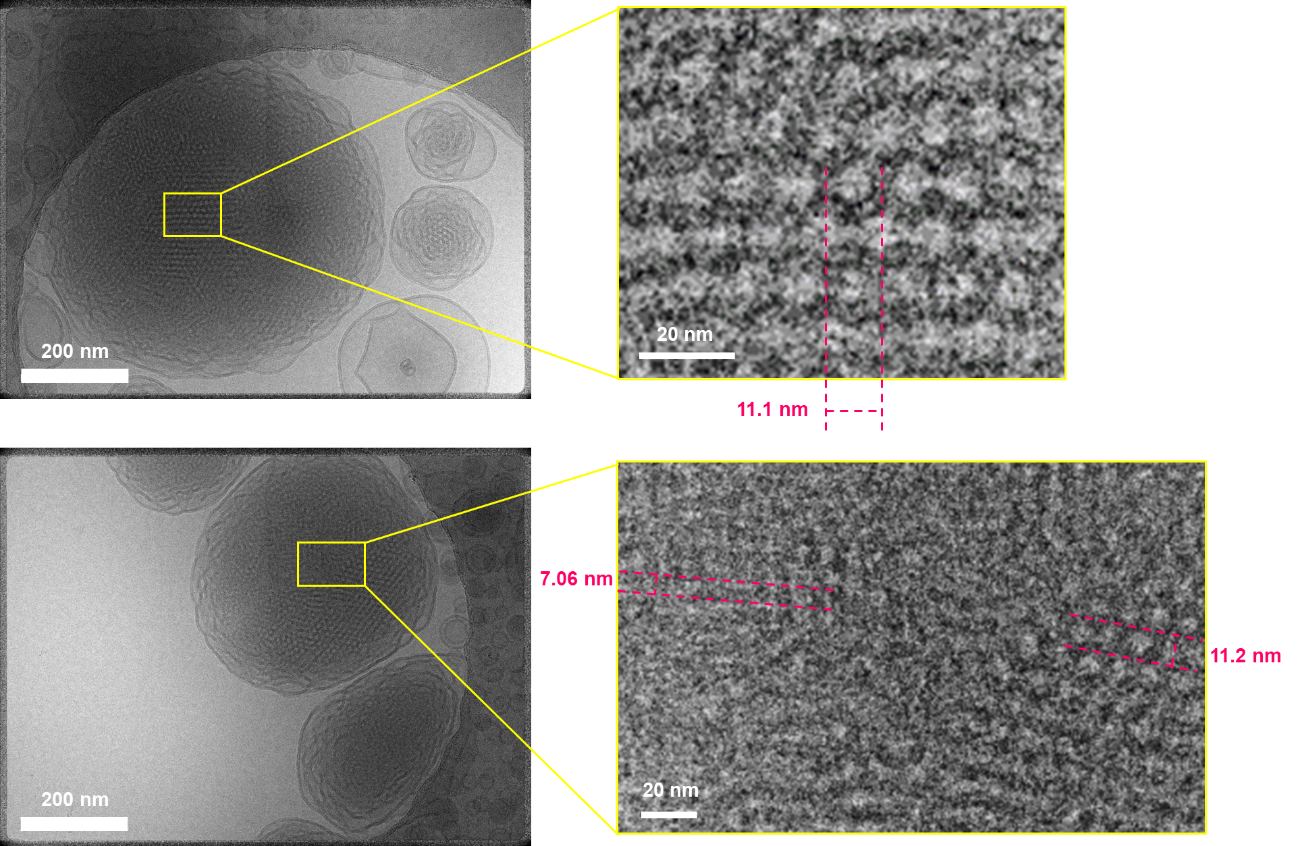


**Figure S2. Measuring the diameter of the unit cubic and hexagonal cells.** The diameter of the unit cubic cell was directly measured from the enlarged image and was determined to be 11.1 nm (upper) and 11.2 nm (lower). The diameter of the unit hexagonal cell was similarly determined to be 7.06 nm (lower).

**Synthesis of DOPE-Cx**

**General Information**

All reactions were monitored by thin-layer chromatography (TLC) on pre-coated TLC plates (Millipore) visualized by UV light (254 nm), phosphomolybdic acid stain, and *p*-anisaldehyde stain. The products were purified using the Biotage Selekt system equipped with an ELSD detector. The separated fractions were analyzed using the Expression CMS TLC-MS system (Advion Interchim Scientific, Ithaca, NY, USA).

All simple chemicals were purchased from commercial sources and were used without further purification.

^1^H and ^13^C NMR spectra were obtained on a JEOL ECZ500R or ECZ400 instrument with tetramethylsilane as the internal standard (0 ppm).

The purity of the final products was analyzed using an LCMS-2050 (Shimazu Corporation) with an ELSD-LT III detector (Shimazu Corporation). Separation was carried out using a Shim-pack Arata C18 Column (pore size: 120 Å, particle size: 5 µm, inner diameter: 50 mm, length: 2.0 mm) and a gradient of 70-98% isopropanol/acetonitrile (2:1) in water with 5 mM of ammonium acetate over 8 min and held at 98% isopropanol/acetonitrile (2:1) with 5 mM of ammonium acetate for 1 min at 0.2 mL/min. The final products were diluted in ethanol to 1,000 ppm and used as the measurement sample. The injection volume was 1 µL and the column temperature was 60 °C.

1. **Synthesis of (R)-2,3-bis(oleoyloxy)propyl (2-((3-butoxy-3-oxopropyl)ammonio)ethyl) phosphate (DOPE-C4).**

1,2-Dioleoyl-sn-3-glycero-phosphoethanolamine (DOPE) (224.4 mg, 0.30 mmol), butyl acrylate (42.3 mg, 0.33 mmol), and diisopropylethylamine (DIPEA) (102 µL, 0.60 mmol) were dissolved in chloroform (1.5 mL) and were stirred at 50 °C for 2 days under an Ar atmosphere. Solvent was removed *in vacuo*. The residue was loaded onto a normal-phase column (Sfär Silica HC D, Biotage) and purified by flash chromatography with a gradient mobile phase of dichloromethane (DCM) and methanol (MeOH). This gave 137.8 mg (52.6%) of **DOPE-C4** as a pale yellow solid.

^1^H NMR (500 MHz, CDCl_3_) σ: 0.88 (t, 6H), 0.92 (t, 3H), 1.22-1.40 (m, 42H), 1.60 (m, 6H), 2.00 (m, 8H), 2.28 (m, 4H), 2.94 (t, 2H), 3.14-3.26 (m, 4H), 4.00-4.19 (m, 5H), 4.24 (br, 2H), 4.37 (m, 1H), 5.22 (m, 1H), 5.33 (m, 4H).


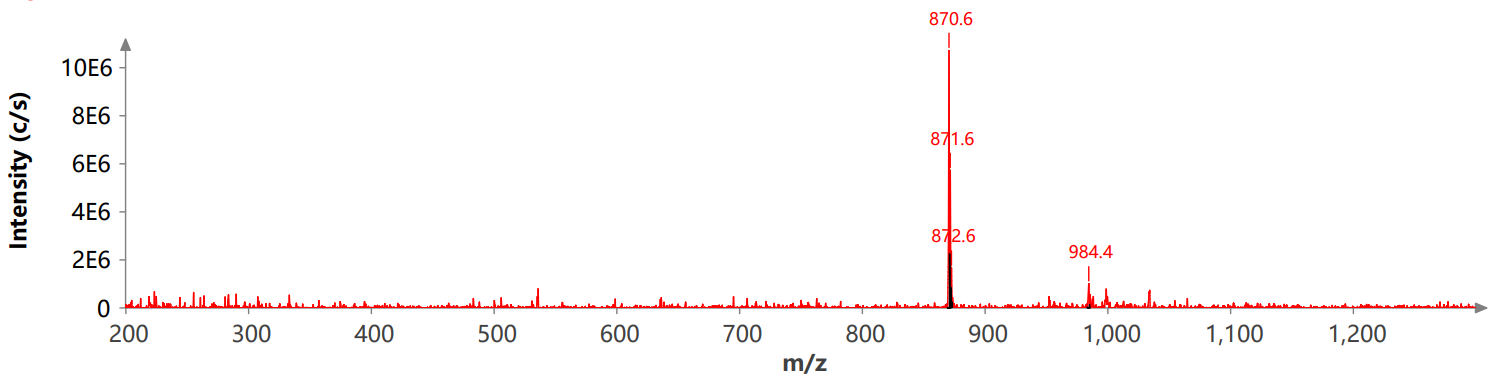


MS (ESI): m/z calculated for C_48_H_89_NO_10_P (M−H)^−^, 870.6; found, 870.6.


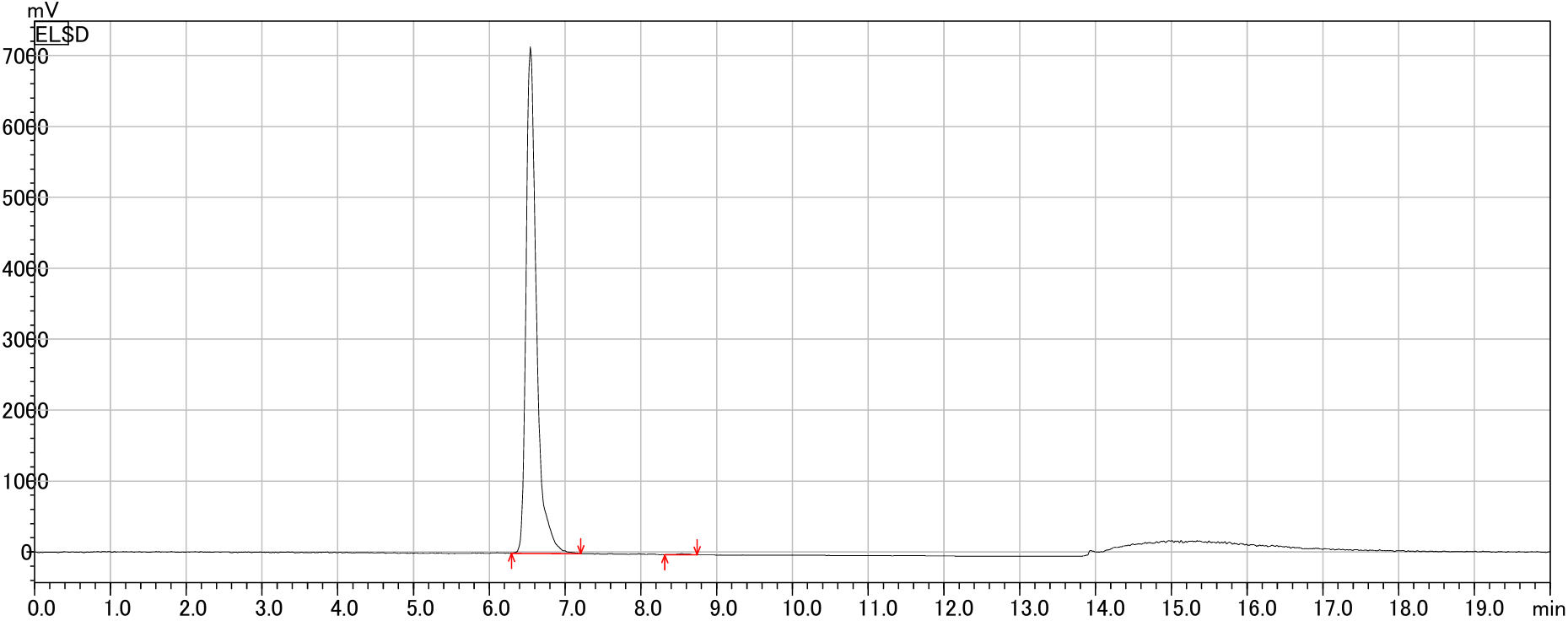


HPLC/ELSD: RT = 6.54 min, purity = 99.8%

1. **Synthesis of (R)-2,3-bis(oleoyloxy)propyl (2-((3-(hexyloxy)-3-oxopropyl)ammonio)ethyl) phosphate (DOPE-C6).**

DOPE (224.4 mg, 0.30 mmol), hexyl acrylate (51.6 mg, 0.33 mmol), and DIPEA (102 µL, 0.60 mmol) were dissolved in chloroform (1.5 mL) and were stirred at 50 °C for 2 days under an Ar atmosphere. Solvent was removed *in vacuo*. The residue was loaded onto a normal-phase column (Sfär Silica HC D, Biotage), and purified by flash chromatography with a gradient mobile phase of DCM and MeOH. This gave 147.4 mg (54.6%) of **DOPE-C6** as a pale yellow solid.

^1^H NMR (500 MHz, CDCl_3_) σ: 0.88 (t, 9H), 1.22-1.37 (m, 46H), 1.60 (m, 6H), 2.00 (m, 8H), 2.28 (m, 4H), 2.98 (t, 2H), 3.14-3.28 (m, 4H), 3.96-4.17 (m, 5H), 4.26 (br, 2H), 4.36 (m, 1H), 5.21 (m, 1H), 5.33 (m, 4H).


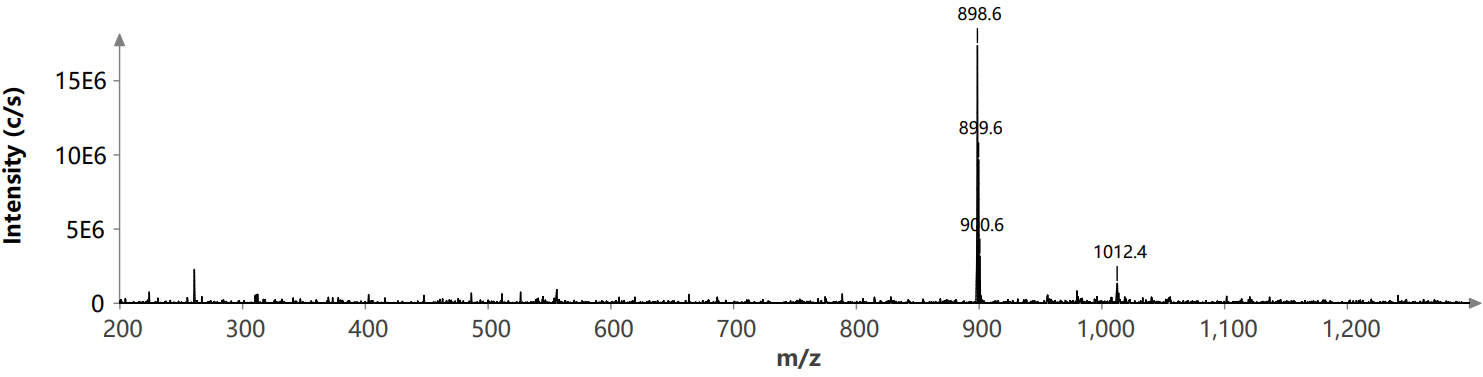


MS (ESI): m/z calculated for C_50_H_93_NO_10_P (M−H)^−^, 898.7; found, 898.6.


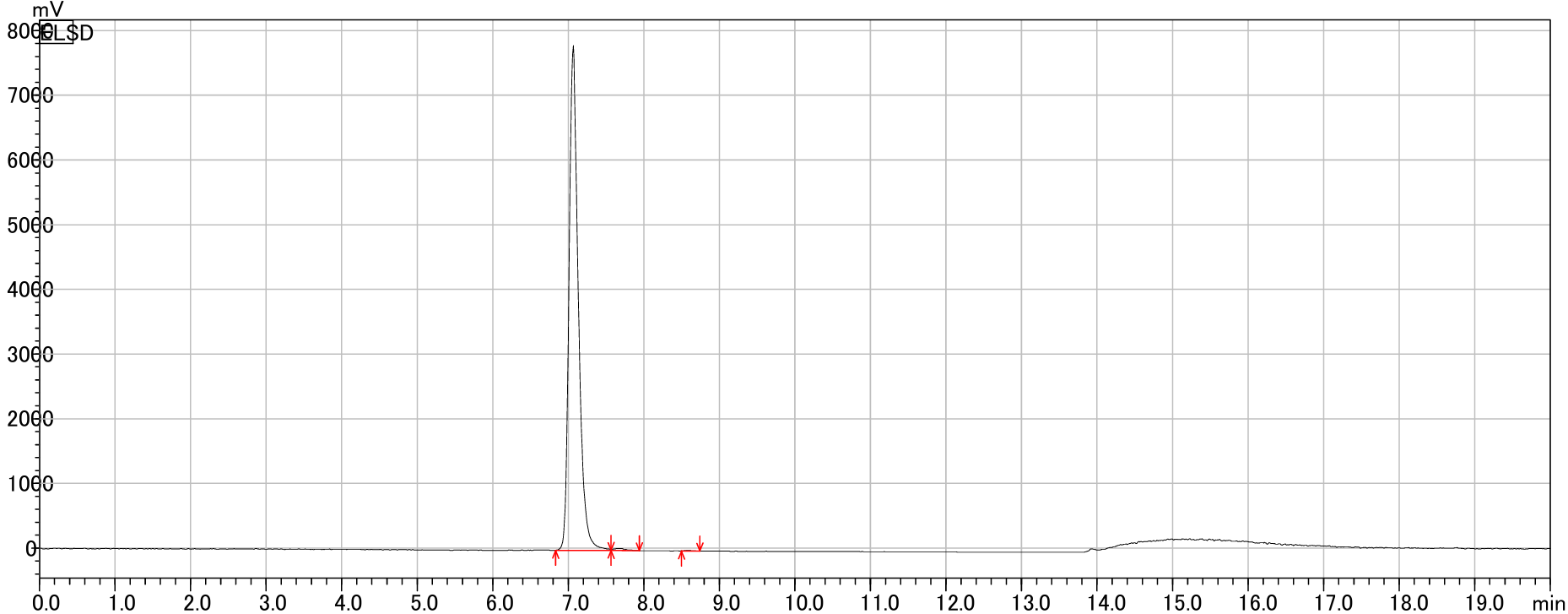


HPLC/ELSD: RT = 7.07 min, purity = 99.3%

**

1. **Synthesis of (R)-2,3-bis(oleoyloxy)propyl (2-((3-(octyloxy)-3-oxopropyl)ammonio)ethyl) phosphate (DOPE-C8).**

DOPE (224.4 mg, 0.30 mmol), n-octyl acrylate (60.8 mg, 0.33 mmol), and triethylamine (TEA) (55.8 µL, 0.40 mmol) were dissolved in chloroform (1.5 mL) and were stirred at 50 °C for 2 days under an Ar atmosphere. Solvent was removed *in vacuo*. The residue was loaded onto a normal-phase column (Sfär Silica HC D, Biotage) and was purified by flash chromatography with a gradient mobile phase of DCM and MeOH. This gave 132.3 mg (47.6%) of **DOPE-C8** as a white solid.

^1^H NMR (500 MHz, CD_3_OD) σ: 0.88 (t, 9H), 1.22-1.40 (m, 50H), 1.62 (m, 6H), 2.02 (m, 8H), 2.32 (m, 4H), 2.80 (t, 2H), 3.33 (t, 4H), 3.99 (t, 2H), 4.05-4.19 (m, 5H), 4.42 (m, 1H), 5.22 (m, 1H), 5.33 (m, 4H).


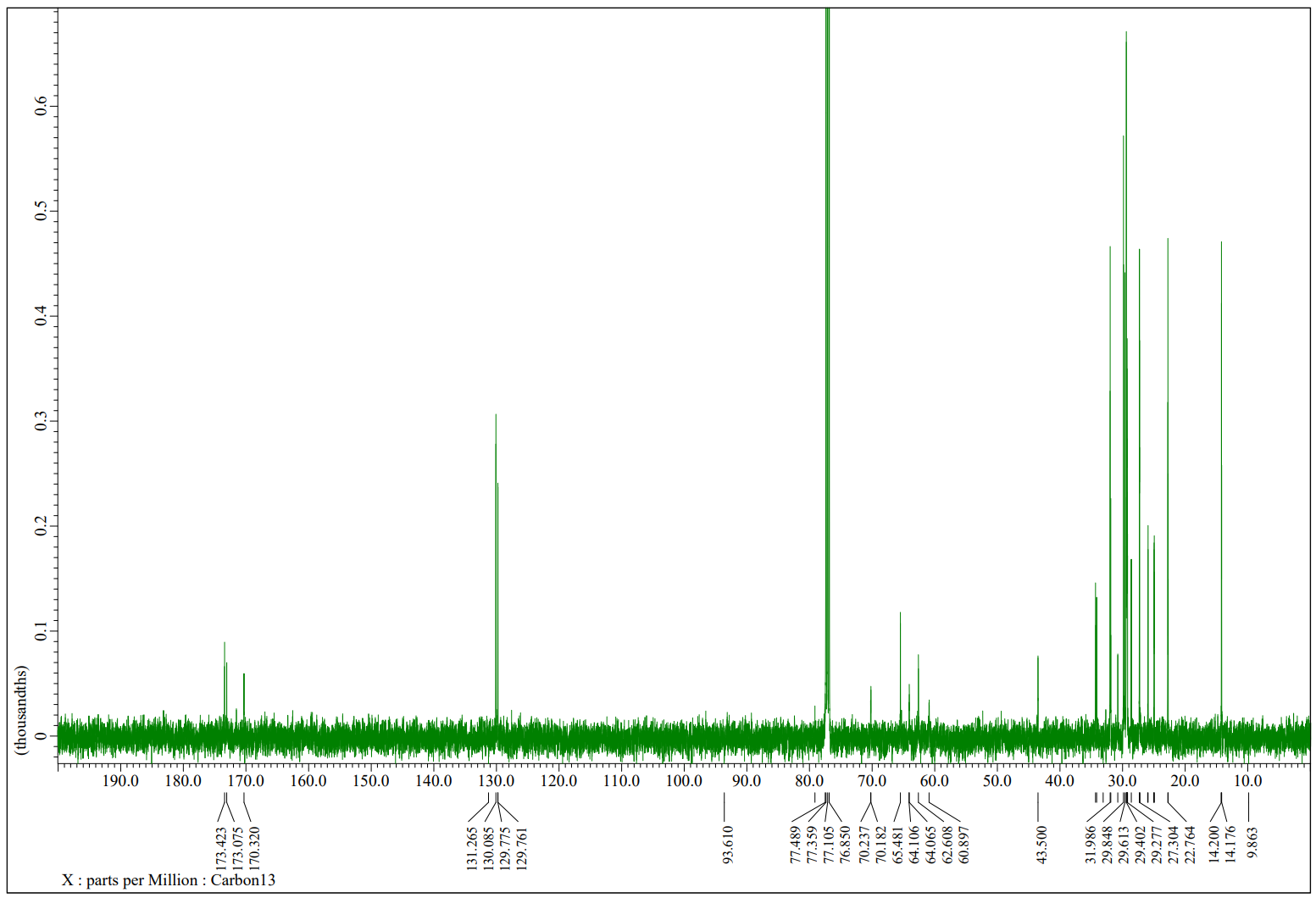


^13^C NMR (500 MHz, CDCl_3_) δ: 14.20, 22.76, 24.94, 25.95, 27.30, 28.55, 29.28, 29.40, 29.61, 29.85, 30.71, 31.91, 31.99, 34.17, 34.32, 43.50, 60.90, 62.61, 64.11, 65.48, 70.18, 79.28, 129.76, 130.09, 170.32, 173.08, 173.42.


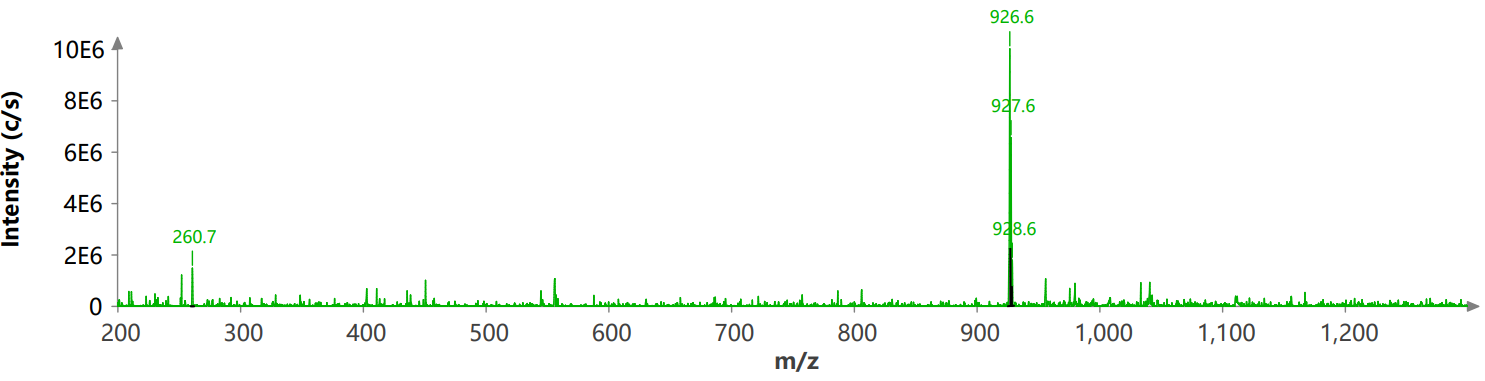


MS (ESI): m/z calculated for C_52_H_97_NO_10_P (M−H)^−^, 926.7; found, 926.6.


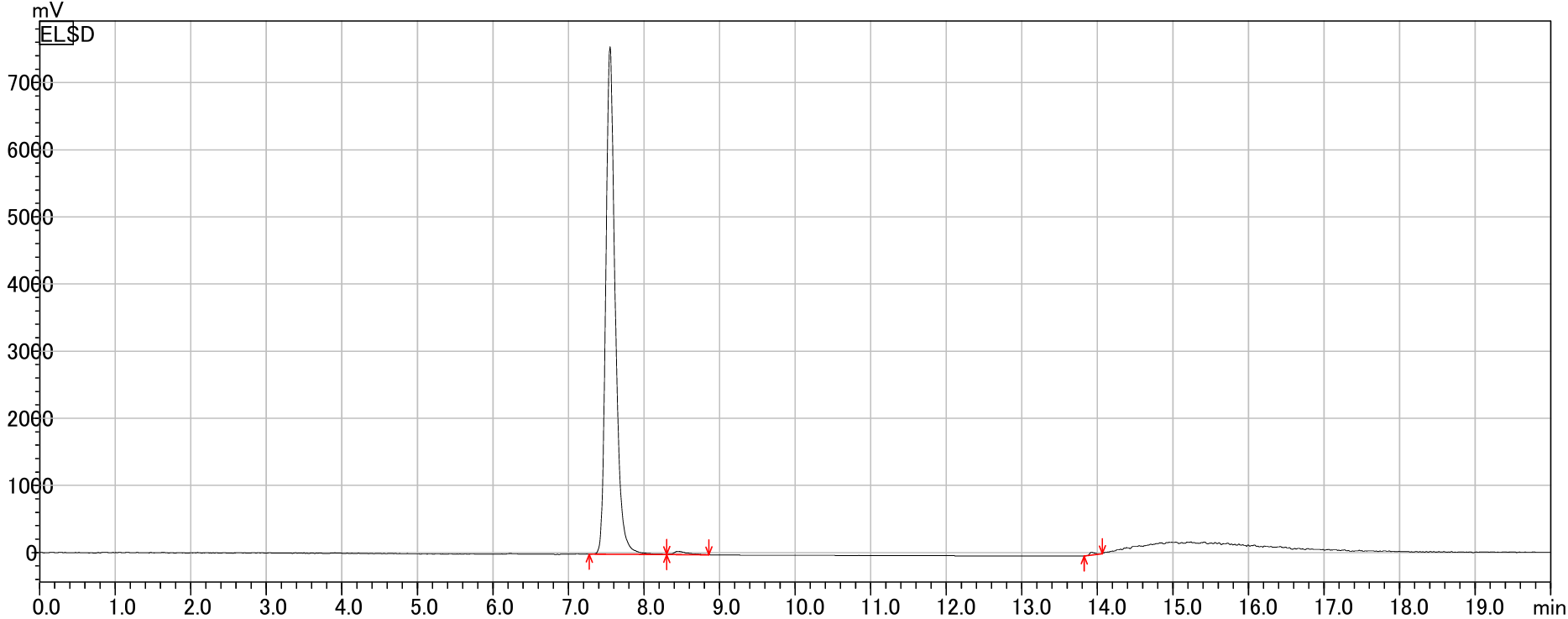


HPLC/ELSD: RT = 7.55 min, purity = 98.8%

1. **Synthesis of (R)-2,3-bis(oleoyloxy)propyl (2-((3-(decyloxy)-3-oxopropyl)ammonio)ethyl) phosphate (DOPE-C10).**

Decanol (158 mg, 1.0 mmol) and TEA (209 µL, 1.50 mmol) were dissolved in anhydrous DCM and then were added with acryloyl chloride (97.0 µL, 1.20 mmol) dropwise to an ice bath. The reaction mixture was stirred at ambient temperature for 2 hours. Solvent was removed *in vacuo*. The residue was dissolved in ethyl acetate (AcOEt) and was washed with water and brine. The organic phase was dried over anhydrous Na_2_SO_4_. After removal of the solvent *in vacuo*, the crude product was dissolved in a mixture of hexane and AcOEt (9:1) and then was passed through a small pad of Wakogel C-100. The filtrate was concentrated *in vacuo*. The crude product (decyl acrylate) was used for the next step without further purification.

DOPE (299.0 mg, 0.40 mmol), decyl acrylate (93.4 mg, 0.44 mmol), and DIPEA (136 µL, 0.80 mmol) were dissolved in chloroform (2.0 mL) and were stirred at 60 °C over night under an Ar atmosphere. Solvent was removed *in vacuo*. The residue was loaded onto a normal-phase column (Sfär Silica HC D, Biotage), and purified by flash chromatography with a gradient mobile phase of DCM and MeOH. This gave 128.8 mg (33.8% from DOPE) of **DOPE-C10** as a pale yellow solid.

^1^H NMR (500 MHz, CDCl_3_) σ: 0.88 (t, 9H), 1.20-1.40 (m, 70H), 1.60 (m, 6H), 2.00 (m, 8H), 2.28 (m, 4H), 2.88 (t, 2H), 3.19 (t, 4H), 3.97-4.26 (m, 7H), 4.36 (m, 1H), 5.22 (m, 1H), 5.33 (m, 4H).


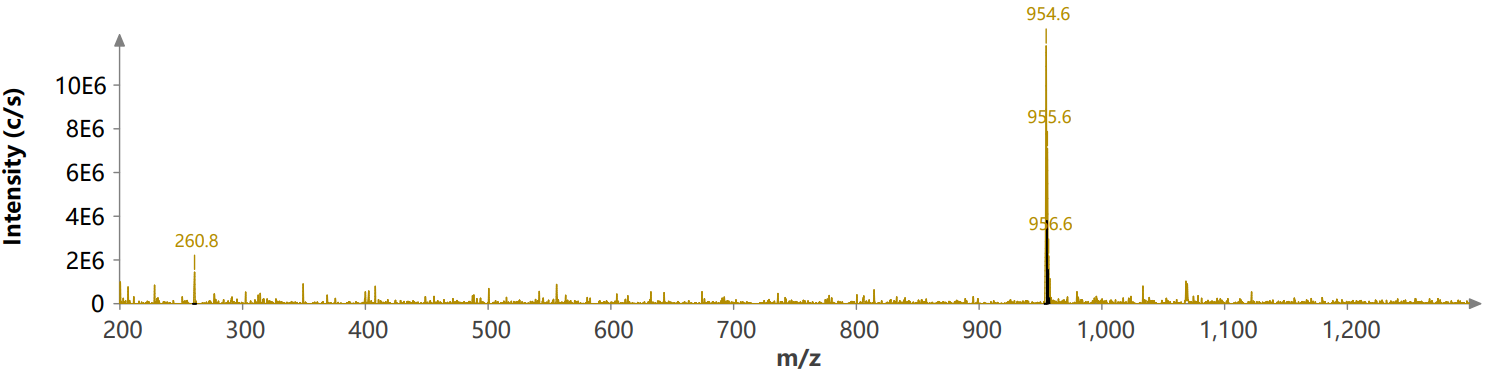


MS (ESI): m/z calculated for C_54_H_101_NO_10_P (M−H)^−^, 954.7; found, 954.6.


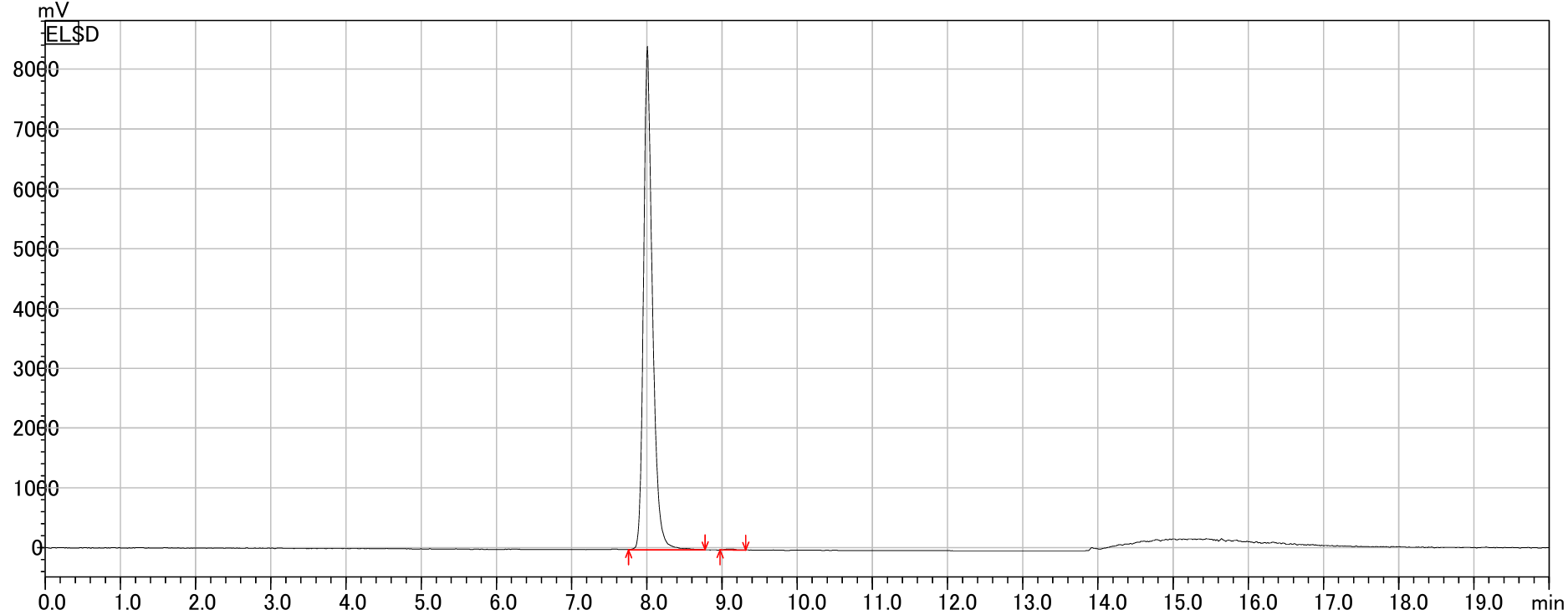


HPLC/ELSD: RT = 8.01 min, purity = 99.8%

1. **Synthesis of (R)-2,3-bis(oleoyloxy)propyl (2-((3-(dodecyloxy)-3-oxopropyl)ammonio)ethyl) phosphate (DOPE-C12).**

DOPE (224.4 mg, 0.30 mmol), dodecyl acrylate (79.3 mg, 0.33 mmol), and TEA (55.8 µL, 0.40 mmol) were dissolved in chloroform (1.5 mL) and were stirred at 50 °C for 2 days under an Ar atmosphere. Solvent was removed *in vacuo*. The residue was loaded onto a normal-phase column (Sfär Silica HC D, Biotage) and purified by flash chromatography with a gradient mobile phase of DCM and MeOH. This gave 159.0 mg (54.0%) of **DOPE-C12** as a pale yellow solid.

^1^H NMR (500 MHz, CDCl_3_) σ: 0.88 (t, 9H), 1.20-1.40 (m, 58H), 1.60 (m, 6H), 2.00 (m, 8H), 2.28 (m, 4H), 2.88 (t, 2H), 3.19 (t, 4H), 3.97-4.26 (m, 7H), 4.39 (m, 1H), 5.22 (m, 1H), 5.33 (m, 4H).


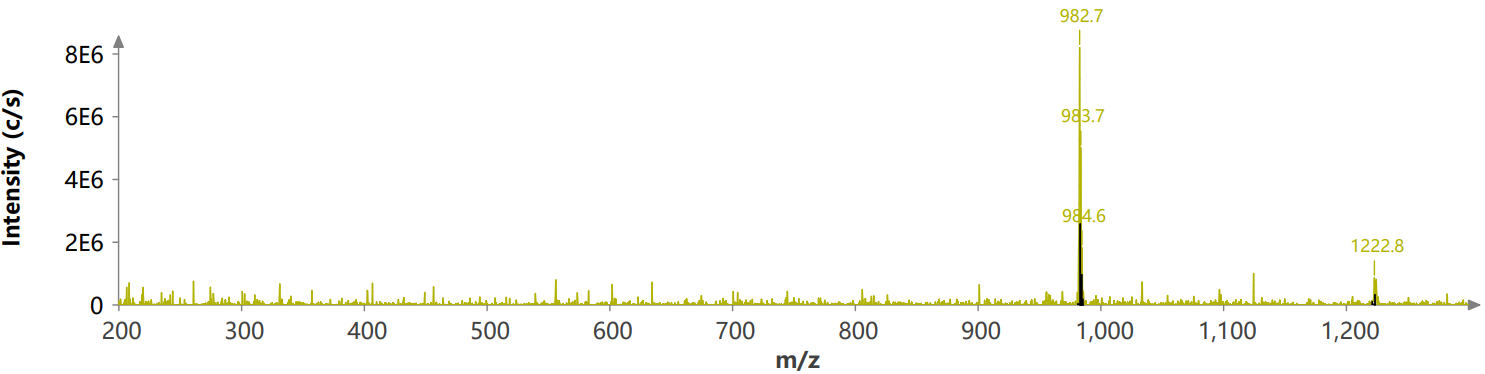


MS (ESI): m/z calculated for C_54_H_101_NO_10_P (M−H)^−^, 982.8; found, 982.7.


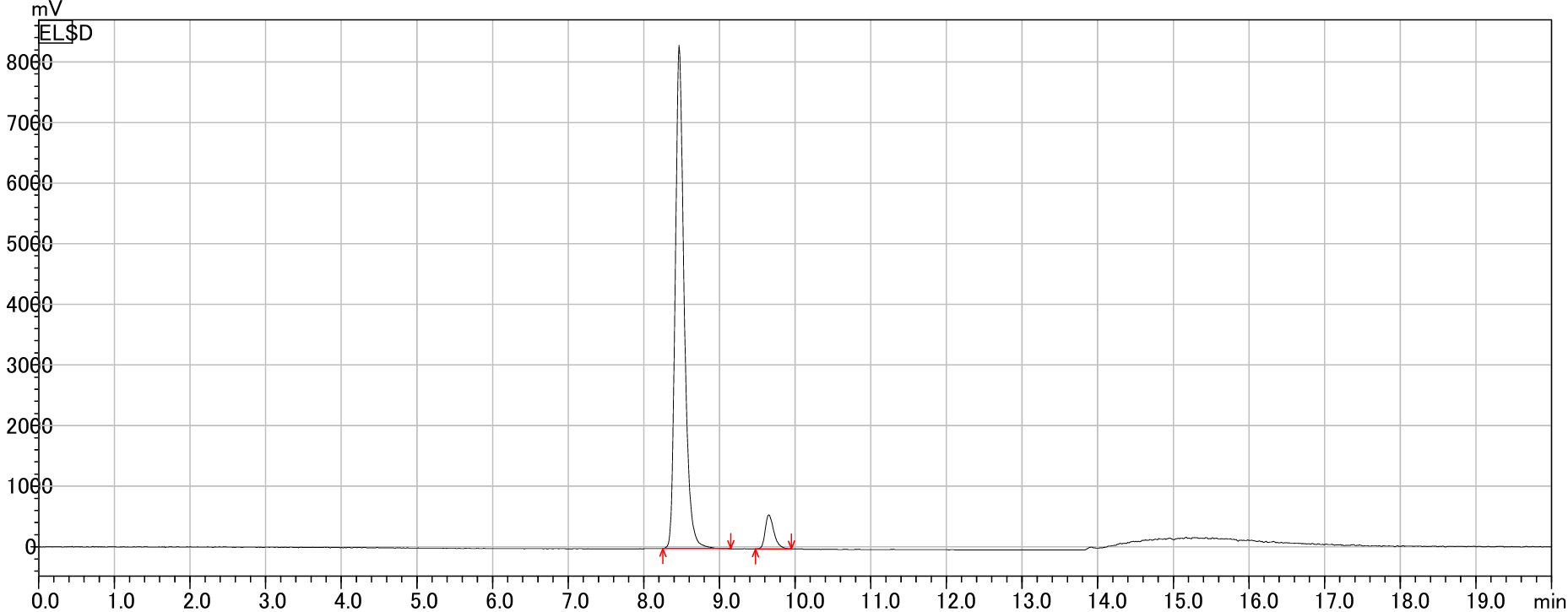


HPLC/ELSD: RT = 8.47 min, purity = 93.8%

1. **Synthesis of (R)-2,3-bis(oleoyloxy)propyl (2-((3-(octadecyloxy)-3-oxopropyl)ammonio)ethyl) phosphate (DOPE-C18).**

DOPE (224.4 mg, 0.30 mmol), stearyl acrylate (107.1 mg, 0.33 mmol), and TEA (55.8 µL, 0.40 mmol) were dissolved in chloroform (1.5 mL) and were stirred at 50 °C for 2 days under an Ar atmosphere. Solvent was removed *in vacuo*. The residue was loaded onto a normal-phase column (Sfär Silica HC D, Biotage) and purified by flash chromatography with a gradient mobile phase of DCM and MeOH. This gave 165.0 mg (51.3%) of **DOPE-C18** as a white solid.

^1^H NMR (500 MHz, CDCl_3_) σ: 0.88 (t, 9H), 1.20-1.40 (m, 70H), 1.60 (m, 6H), 2.00 (m, 8H), 2.28 (m, 4H), 2.88 (t, 2H), 3.19 (t, 4H), 3.97-4.26 (m, 7H), 4.36 (m, 1H), 5.22 (m, 1H), 5.33 (m, 4H).

1. **Synthesis of (R)-2,3-bis(oleoyloxy)propyl (2-((3-(((Z)-octadec-9-en-1-yl)oxy)-3-oxopropyl)ammonio)ethyl) phosphate (DOPE-C18:1).**

Oleyl alcohol (269 mg, 1.0 mmol) and TEA (209 µL, 1.50 mmol) were dissolved in anhydrous DCM and then were added with acryloyl chloride (80.8 µL, 1.0 mmol) dropwise to an ice bath. The reaction mixture was stirred at ambient temperature for 1 hour. Solvent was removed *in vacuo*. The residue was suspended in hexane and was filtered. The filtrate was loaded onto a normal-phase column (Sfär Silica HC D, Biotage) and purified by flash chromatography with a gradient mobile phase of hexane and AcOEt. This gave oleyl acrylate as a colorless oil.

DOPE (299.0 mg, 0.40 mmol), oleyl acrylate (142.0 mg, 0.44 mmol), and DIPEA (136 µL, 0.80 mmol) were dissolved in chloroform (2.0 mL) and were stirred at 60 °C over night under an Ar atmosphere. Solvent was removed *in vacuo*. The residue was loaded onto a normal-phase column (Sfär Silica HC D, Biotage) and purified by flash chromatography with a gradient mobile phase of DCM and MeOH. This gave 152.4 mg (35.8% from DOPE) of **DOPE-C18:1** as a yellow solid.

^1^H NMR (500 MHz, CDCl_3_) σ: 0.88 (t, 9H), 1.20-1.40 (m, 62H), 1.60 (m, 6H), 2.00 (m, 12H), 2.29 (m, 4H), 2.89 (br, 2H), 3.19 (br, 4H), 3.97-4.26 (m, 7H), 4.38 (m, 1H), 5.22 (m, 1H), 5.33 (m, 6H).


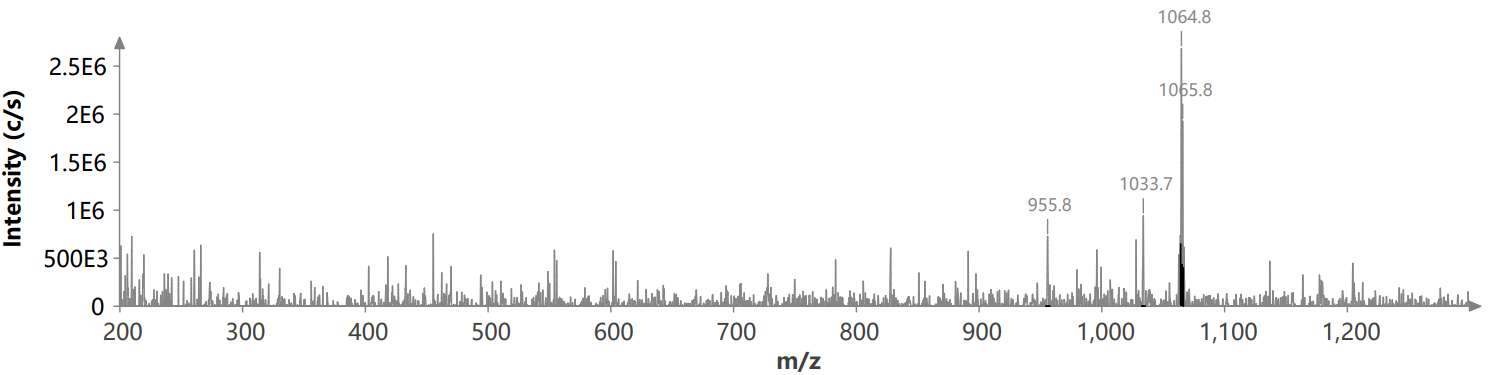


MS (ESI): m/z calculated for C_62_H_115_NO_10_P (M−H)^−^, 1064.8; found, 1064.8.


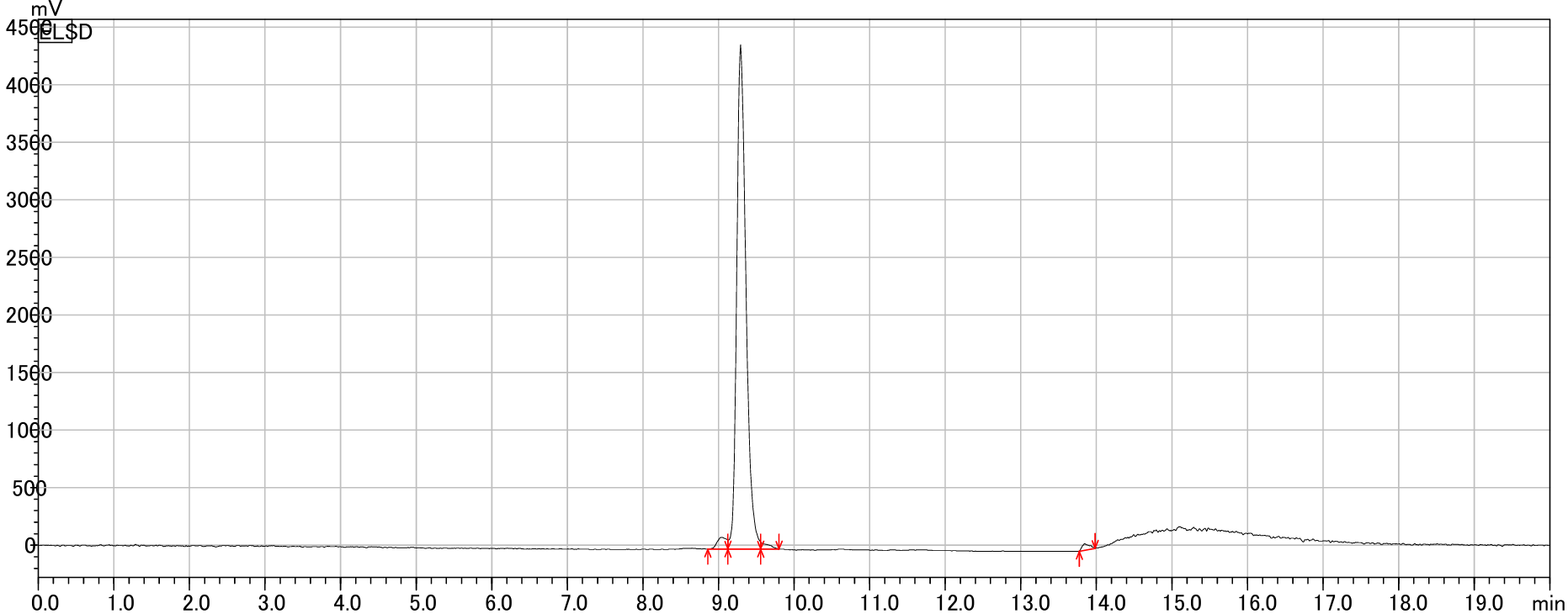


HPLC/ELSD: RT = 9.29 min, purity = 95.6%

1. **Synthesis of (R)-2,3-bis(oleoyloxy)propyl (2-((3-((2-methylpentyl)oxy)-3-oxopropyl)ammonio)ethyl) phosphate (DOPE-C5_β-1).**

2-Methyl-1-pentanol (102.2 mg, 1.0 mmol) and TEA (209 µL, 1.50 mmol) were dissolved in anhydrous DCM (4 mL) and then were added with acryloyl chloride (80.8 µL, 1.0 mmol) dropwise to an ice bath. The reaction mixture was stirred at ambient temperature for 2 hours. Solvent was removed *in vacuo*. The residue was suspended in hexane/AcOEt (9:1) and was passed through a silica pad. The filtrate was concentrated *in vacuo*. The crude product (2-methylpentyl acrylate) was used for the next reaction without further purification.

DOPE (598.5 mg, 0.80 mmol), the crude product (2-methylpentyl acrylate) (107.1 mg), and TEA (209 µL, 1.50 mol) were dissolved in chloroform (4 mL) and were stirred at 60 °C for 2 days under an Ar atmosphere. The reaction mixture was loaded onto a normal-phase column (Sfär Silica HC D, Biotage), and purified by flash chromatography with a gradient mobile phase of DCM and MeOH. This gave 284.3 mg (39.5%) of **DOPE-C5_β-1** as a yellow viscous oil.

^1^H NMR (500 MHz, CD_3_OD) σ: 0.88 (t, 12H), 1.20-1.43 (m, 44H), 1.60 (m, 4H), 1.80 (m, 1H), 2.00 (m, 8H), 2.31 (m, 4H), 2.80 (t, 2H), 3.19 (t, 2H), 3.33 (t, 2H), 3.90-4.20 (m, 7H), 4.42 (m, 1H), 5.22 (m, 1H), 5.33 (m, 4H).


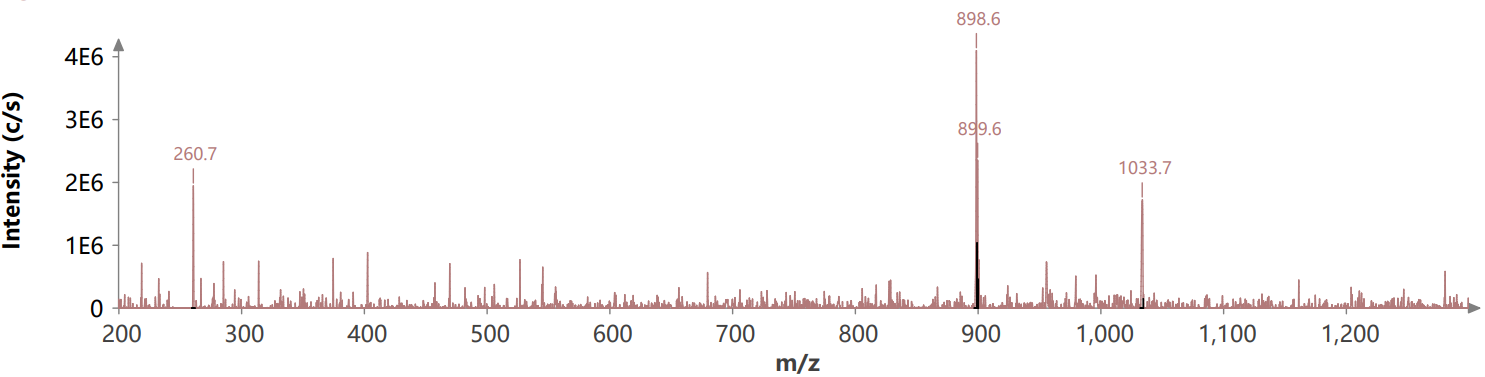


MS (ESI): m/z calculated for C_50_H_93_NO_10_P (M−H)^−^, 898.7; found, 898.6.


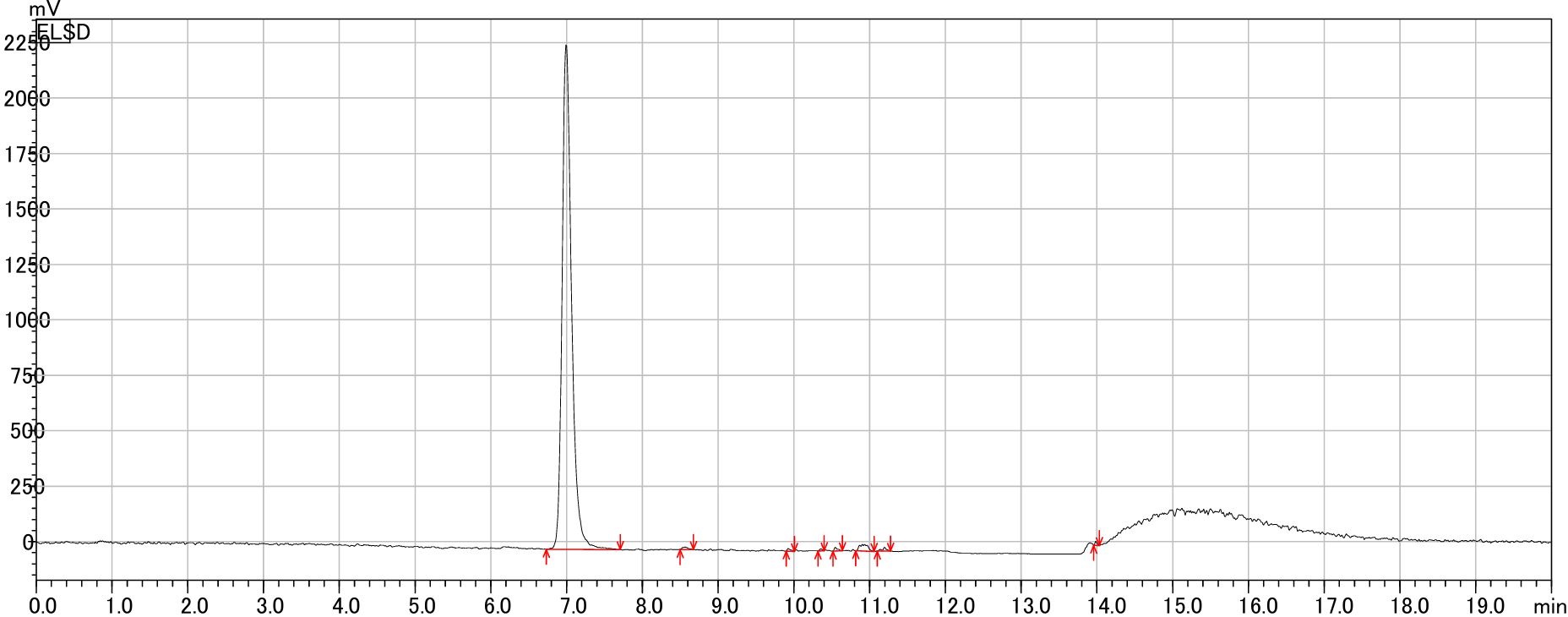


HPLC/ELSD: RT = 7.00 min, purity = 97.5%

1. **Synthesis of (R)-2,3-bis(oleoyloxy)propyl (2-((3-((2-ethylhexyl)oxy)-3-oxopropyl)ammonio)ethyl) phosphate (DOPE-C6_β-2).**

2-Ethyl-1-hexanol (130.2 mg, 1.0 mmol) and TEA (209 µL, 1.50 mmol) were dissolved in anhydrous DCM (4 mL) and then were added with acryloyl chloride (80.8 µL, 1.0 mmol) dropwise to an ice bath. The reaction mixture was stirred at ambient temperature for 2 hours. Solvent was removed *in vacuo*. The residue was suspended in hexane/AcOEt (9:1) and was passed through a silica pad. The filtrate was concentrated *in vacuo*. The crude product (2-ethylhexyl acrylate) was used for the next reaction without further purification.

DOPE (598.5 mg, 0.80 mmol), the crude product (2-ethylhexyl acrylate) (158.0 mg), and TEA (209 µL, 1.50 mol) were dissolved in chloroform (4 mL) and were stirred at 60 °C for 2 days under an Ar atmosphere. The reaction mixture was loaded onto a normal-phase column (Sfär Silica HC D, Biotage), and purified by flash chromatography with a gradient mobile phase of DCM and MeOH. This gave 524.3 mg (70.6%) of **DOPE-C6_β-2** as a yellow viscous oil.

^1^H NMR (500 MHz, CD_3_OD) σ: 0.88 (t, 12H), 1.21-1.43 (m, 48H), 1.60 (m, 5H), 2.01 (m, 8H), 2.31 (m, 4H), 2.80 (t, 2H), 3.19 (t, 2H), 3.33 (t, 2H), 3.96-4.18 (m, 7H), 4.42 (m, 1H), 5.22 (m, 1H), 5.33 (m, 4H).


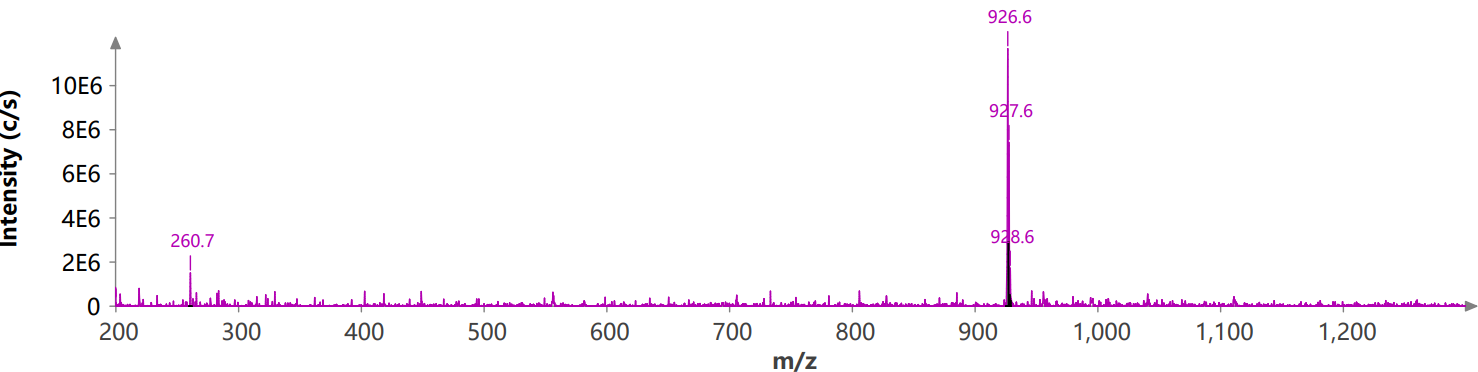


MS (ESI): m/z calculated for C_52_H_97_NO_10_P (M−H)^−^, 926.7; found, 926.6.


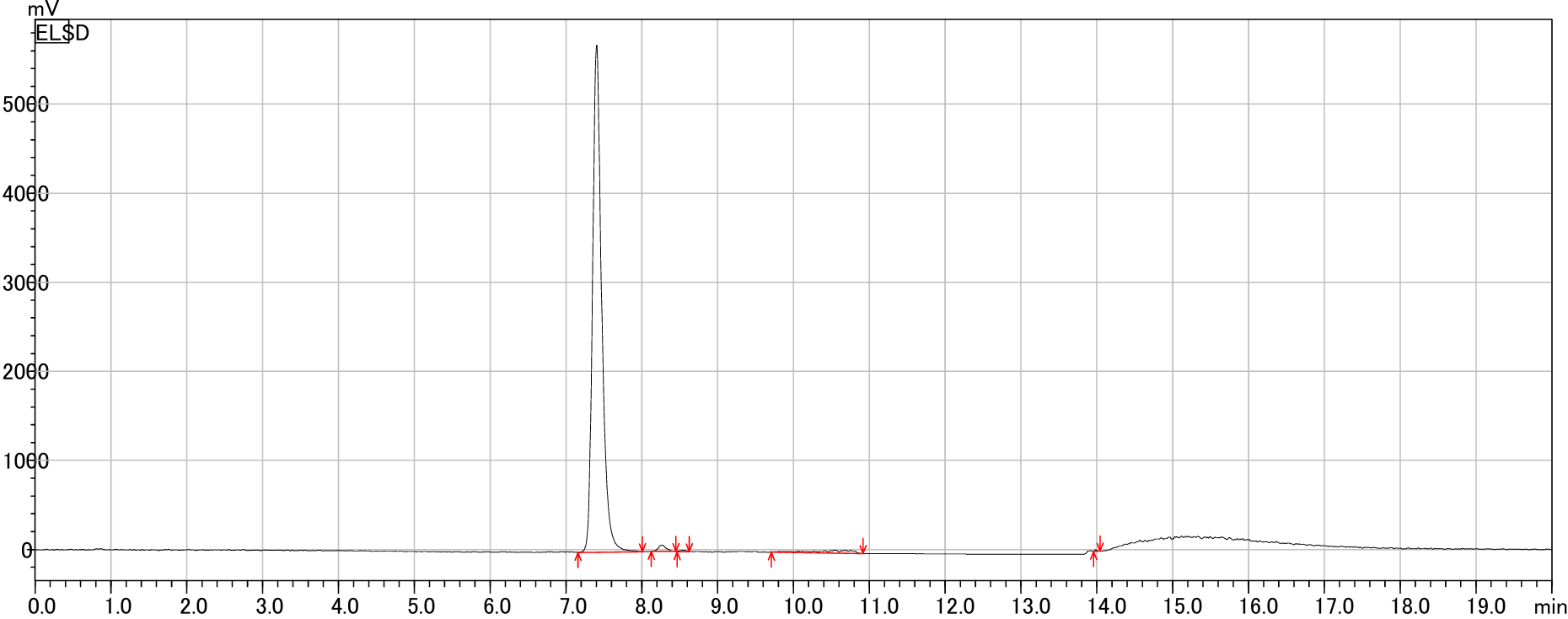


HPLC/ELSD: RT = 7.41 min, purity = 96.3%

1. **Synthesis of (R)-2,3-bis(oleoyloxy)propyl (2-((3-oxo-3-((2-propylheptyl)oxy)propyl)ammonio)ethyl) phosphate (DOPE-C7_β-3).**

2-Propyl-1-heptan-1-ol (158.3 mg, 1.0 mmol) and TEA (209 µL, 1.50 mmol) were dissolved in anhydrous DCM (4 mL) and then were added with acryloyl chloride (80.8 µL, 1.0 mmol) dropwise to an ice bath. The reaction mixture was stirred at ambient temperature for 2 hours. Solvent was removed *in vacuo*. The residue was suspended in hexane/AcOEt (9:1) and was passed through a silica pad. The filtrate was concentrated *in vacuo*. The crude product (2-propylheptyl acrylate) was used for the next reaction without further purification.

DOPE (598.5 mg, 0.80 mmol), the crude product (2-propylheptyl acrylate) (152.3 mg), and TEA (209 µL, 1.50 mol) were dissolved in chloroform (4 mL) and were stirred at 60 °C for 2 days under an Ar atmosphere. The reaction mixture was loaded onto a normal-phase column (Sfär Silica HC D, Biotage), and purified by flash chromatography with a gradient mobile phase of DCM and MeOH. This gave 307 mg (40.1%) of **DOPE-C7_β-3** as a pale yellow viscous oil.

^1^H NMR (500 MHz, CD_3_OD) σ: 0.88 (t, 12H), 1.21-1.40 (m, 52H), 1.60 (m, 4H), 1.66 (m, 1H), 2.01 (m, 8H), 2.31 (m, 4H), 2.80 (t, 2H), 3.33 (t, 4H), 3.96-4.18 (m, 7H), 4.42 (m, 1H), 5.22 (m, 1H), 5.33 (m, 4H).


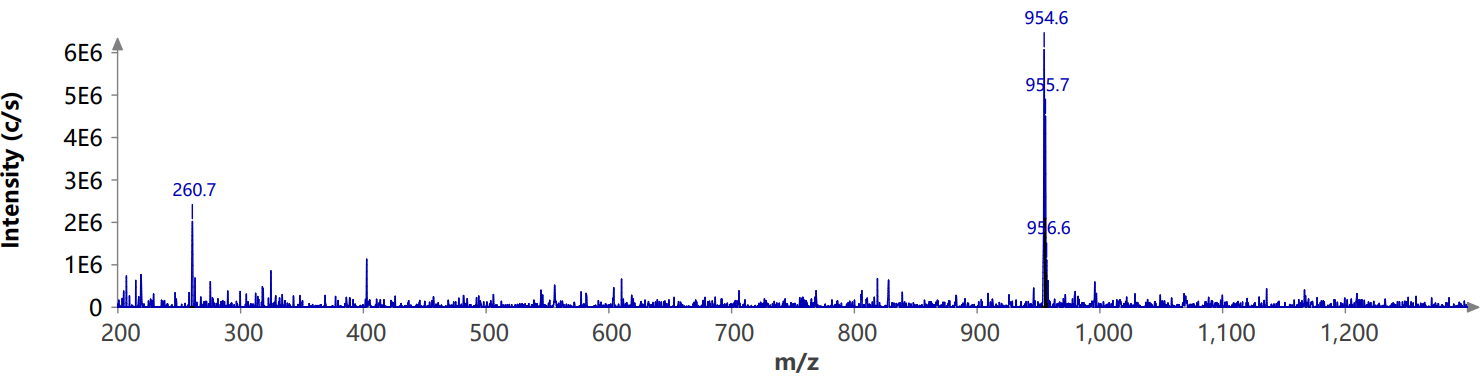


MS (ESI): m/z calculated for C_54_H_101_NO_10_P (M−H)^−^, 954.7; found, 954.6.


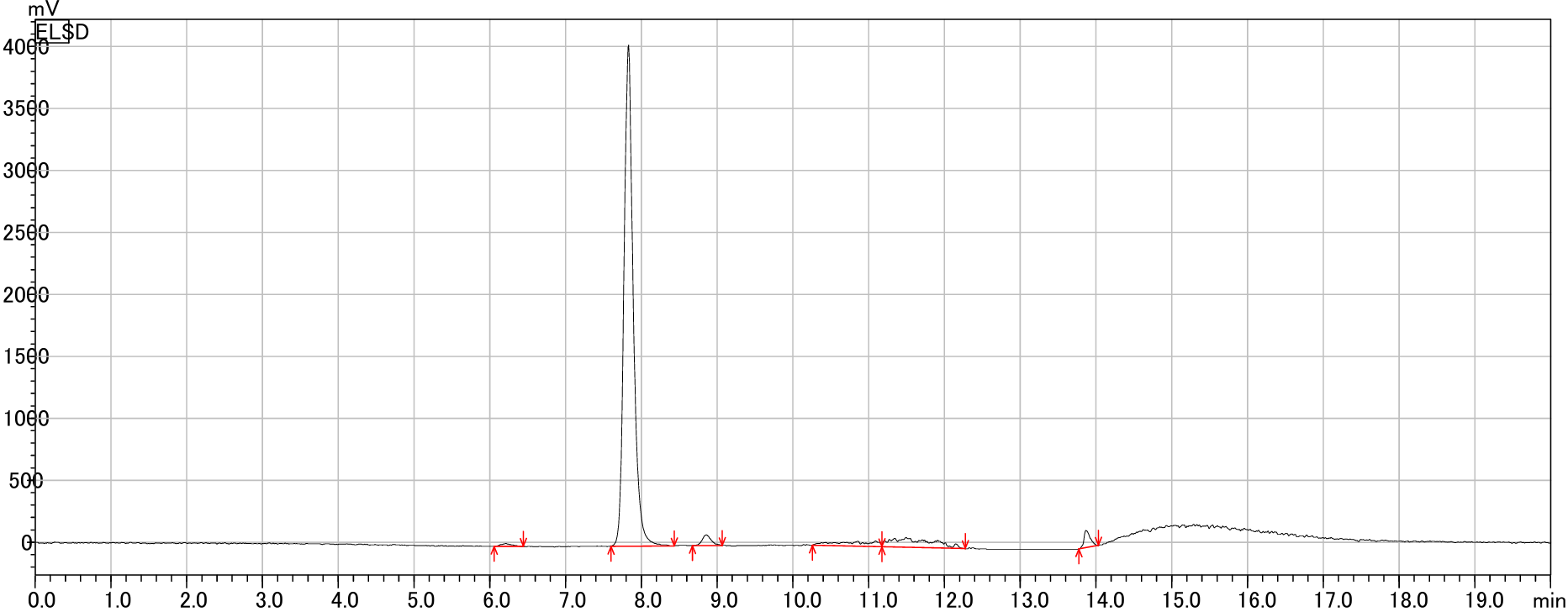


HPLC/ELSD: RT = 7.83 min, purity = 85.4%

1. **Synthesis of (R)-2,3-bis(oleoyloxy)propyl (2-((3-((2-butyloctyl)oxy)-3-oxopropyl)ammonio)ethyl) phosphate (DOPE-C8_β-4).**

2-Butyl-1-n-octanol (186.3 mg, 1.0 mmol) and TEA (209 µL, 1.50 mmol) were dissolved in anhydrous DCM (4 mL) and then were added with acryloyl chloride (80.8 µL, 1.0 mmol) dropwise to an ice bath. The reaction mixture was stirred at ambient temperature for 2 hours. Solvent was removed *in vacuo*. The residue was suspended in hexane/AcOEt (9:1) and was passed through a silica pad. The filtrate was concentrated *in vacuo*. The crude product (2-butyloctyl acrylate) was used for the next reaction without further purification.

DOPE (598.5 mg, 0.80 mmol), the crude product (2-butyloctyl acrylate) (190.1 mg), and TEA (209 µL, 1.50 mol) were dissolved in chloroform (4 mL) and were stirred at 60 °C for 2 days under an Ar atmosphere. The reaction mixture was loaded onto a normal-phase column (Sfär Silica HC D, Biotage), and purified by flash chromatography with a gradient mobile phase of DCM and MeOH. This gave 303 mg (38.5%) of **DOPE-C8_β-4** as a pale yellow viscous oil.

^1^H NMR (500 MHz, CD_3_OD) σ: 0.88 (t, 12H), 1.21-1.39 (m, 56H), 1.60 (m, 4H), 1.65 (m, 1H), 2.01 (m, 8H), 2.31 (m, 4H), 2.80 (t, 2H), 3.33 (t, 4H), 3.96-4.18 (m, 7H), 4.42 (m, 1H), 5.22 (m, 1H), 5.33 (m, 4H).


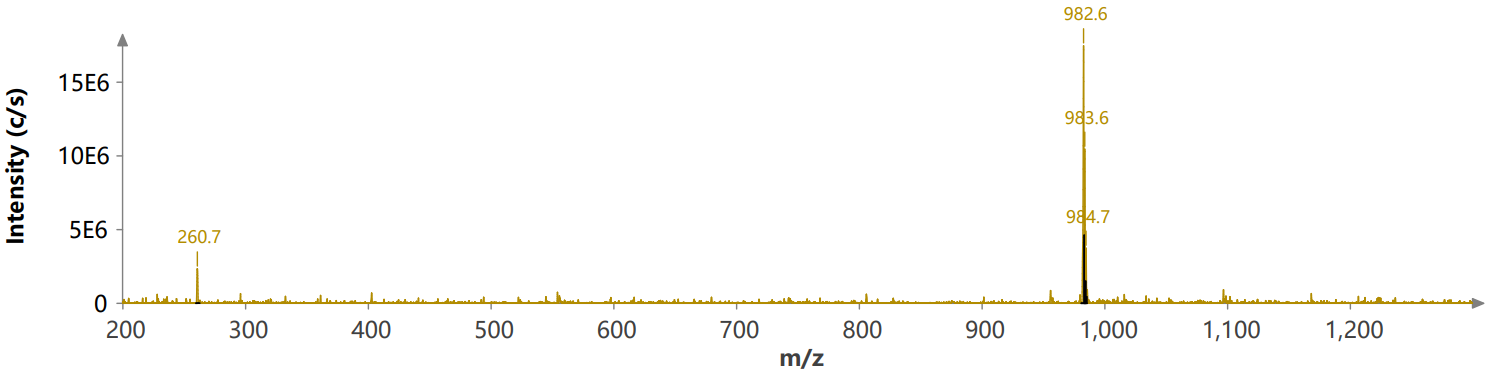


MS (ESI): m/z calculated for C_56_H_105_NO_10_P (M−H)^−^, 982.7; found, 982.6.


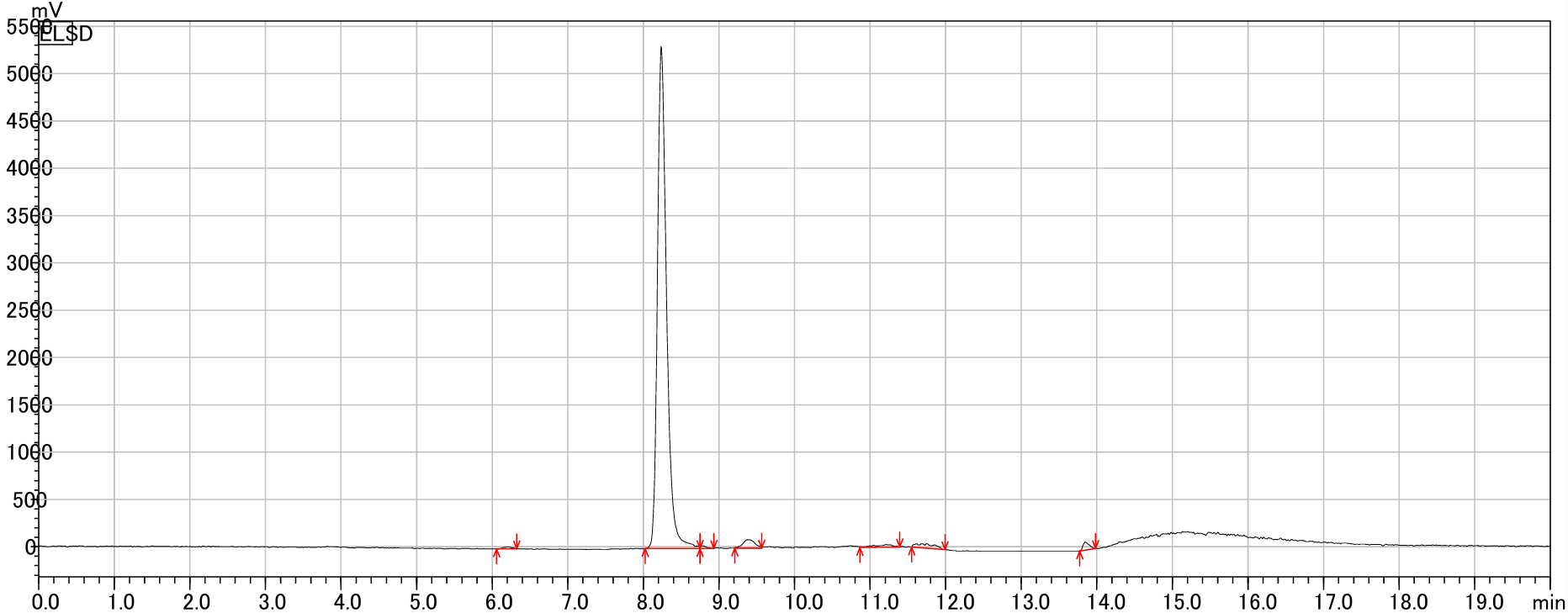


HPLC/ELSD: RT = 8.24 min, purity = 93.6%

1. **Synthesis of (R)-2,3-bis(oleoyloxy)propyl (2-((3-((3,7-dimethyloct-6-en-1-yl)oxy)-3-oxopropyl)ammonio)ethyl) phosphate (DOPE-Cit).**

β-Citronellol (156.3 mg, 1.0 mmol) and TEA (209 µL, 1.50 mmol) were dissolved in anhydrous DCM (4 mL) and then were added with acryloyl chloride (80.8 µL, 1.0 mmol) dropwise to an ice bath. The reaction mixture was stirred at ambient temperature for 2 hours. Solvent was removed *in vacuo*. The residue was suspended in hexane/AcOEt (9:1) and was passed through a silica pad. The filtrate was concentrated *in vacuo*. The crude product (3,7-dimethyloct-6-en-1-yl acrylate) was used for the next reaction without further purification.

DOPE (598.5 mg, 0.80 mmol), the crude product (3,7-dimethyloct-6-en-1-yl acrylate) (150.1 mg), and TEA (209 µL, 1.50 mol) were dissolved in chloroform (4 mL) and were stirred at 60 °C for 2 days under an Ar atmosphere. The reaction mixture was loaded onto a normal-phase column (Sfär Silica HC D, Biotage) and purified by flash chromatography with a gradient mobile phase of DCM and MeOH. This gave 98.6 mg (12.9%) of **DOPE-Cit** as a pale yellow solid.

^1^H NMR (500 MHz, CD_3_OD) σ: 0.88 (t, 9H), 122-1.39 (m, 43H), 1.60 (m, 7H), 1.65 (s, 3H), 2.01 (m, 10H), 2.31 (m, 4H), 2.80 (t, 2H), 3.19 (t, 2H), 3.33 (t, 4H), 3.96-4.20 (m, 7H), 4.42 (m, 1H), 5.09 (t, 1H), 5.22 (m, 1H), 5.33 (m, 4H).


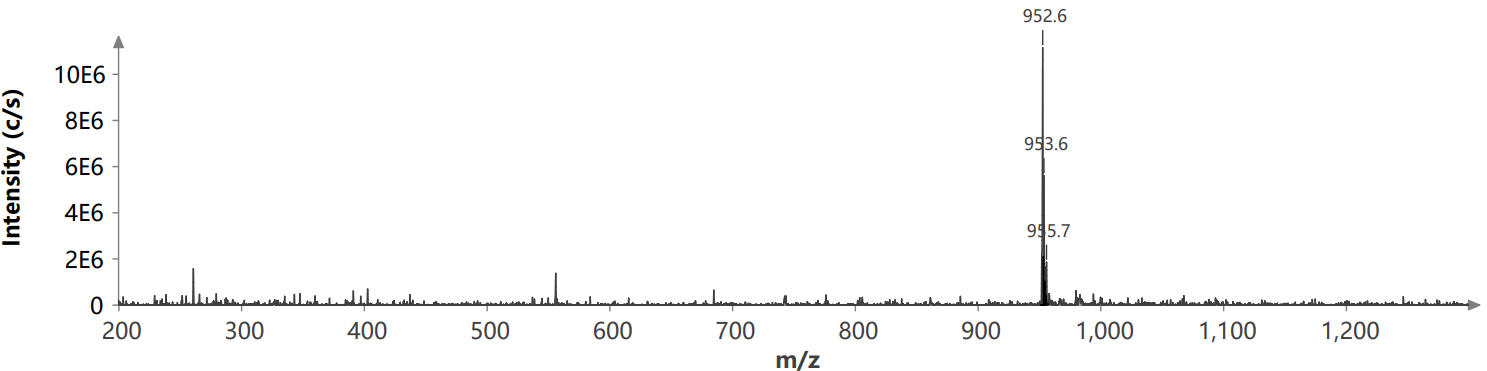


MS (ESI): m/z calculated for C_54_H_99_NO_10_P (M−H)^−^, 952.7; found, 952.6.


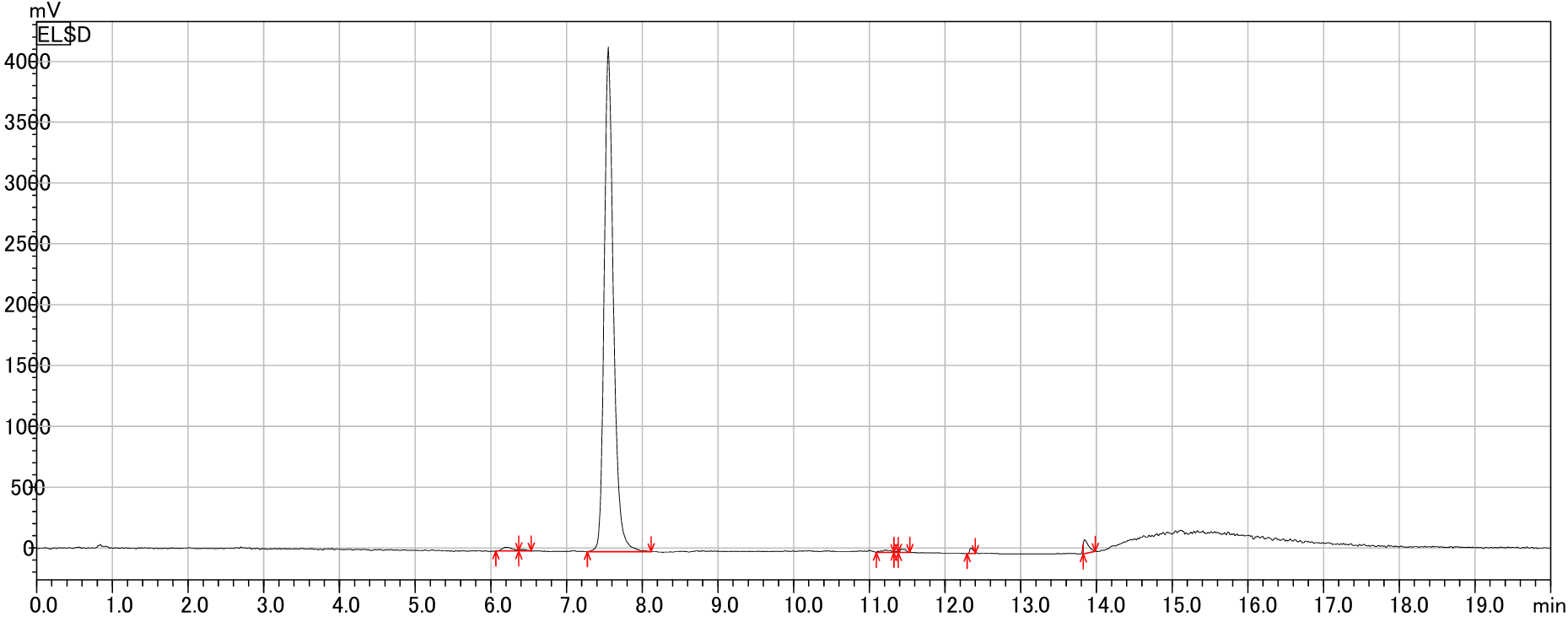


HPLC/ELSD: RT = 7.55 min, purity = 96.3%

1. **Synthesis of (R)-2,3-bis(oleoyloxy)propyl (2-((3-oxo-3-(pentan-3-yloxy)propyl)ammonio)ethyl) phosphate (DOPE-C3_α-2).**

3-Pentanol (88.2 mg, 1.0 mmol) and TEA (209 µL, 1.50 mmol) were dissolved in anhydrous DCM (4 mL) and then were added with acryloyl chloride (80.8 µL, 1.0 mmol) dropwise to an ice bath. The reaction mixture was stirred at ambient temperature for 2 hours. Solvent was removed *in vacuo*. The residue was suspended in hexane/AcOEt (9:1) and was passed through a silica pad. The filtrate was concentrated *in vacuo*. The crude product (pentan-3-yl acrylate) was used for the next reaction without further purification.

DOPE (598.5 mg, 0.80 mmol), the crude product (pentan-3-yl acrylate) (135 mg), and TEA (209 µL, 1.50 mol) were dissolved in chloroform (4 mL) and were stirred at 60 °C for 2 days under an Ar atmosphere. The reaction mixture was loaded onto a normal-phase column (Sfär Silica HC D, Biotage), and purified by flash chromatography with a gradient mobile phase of DCM and MeOH. This gave 262 mg (36.9%) of **DOPE-C3_α-2** as a yellow viscous oil.

^1^H NMR (500 MHz, CD_3_OD) σ: 0.88 (t, 12H), 1.21-1.38 (m, 40H), 1.60 (m, 8H), 2.01 (m, 8H), 2.31 (m, 4H), 2.80 (t, 2H), 3.33 (t, 4H), 3.96-4.18 (m, 7H), 4.42 (m, 1H), 4.80 (m, 1H), 5.22 (m, 1H), 5.33 (m, 4H).


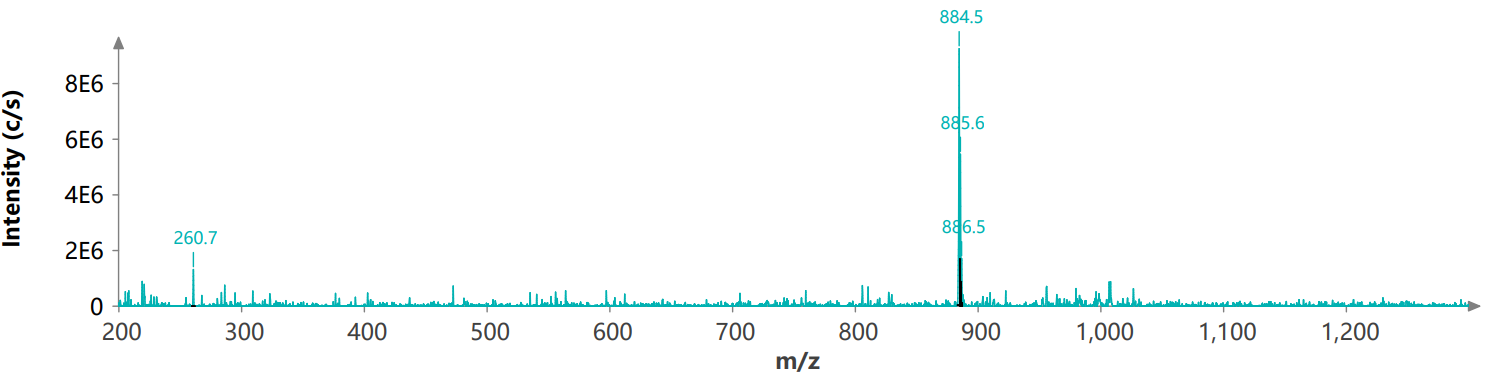


MS (ESI): m/z calculated for C_49_H_92_NO_10_P (M−H)^−^, 884.7; found, 884.5.


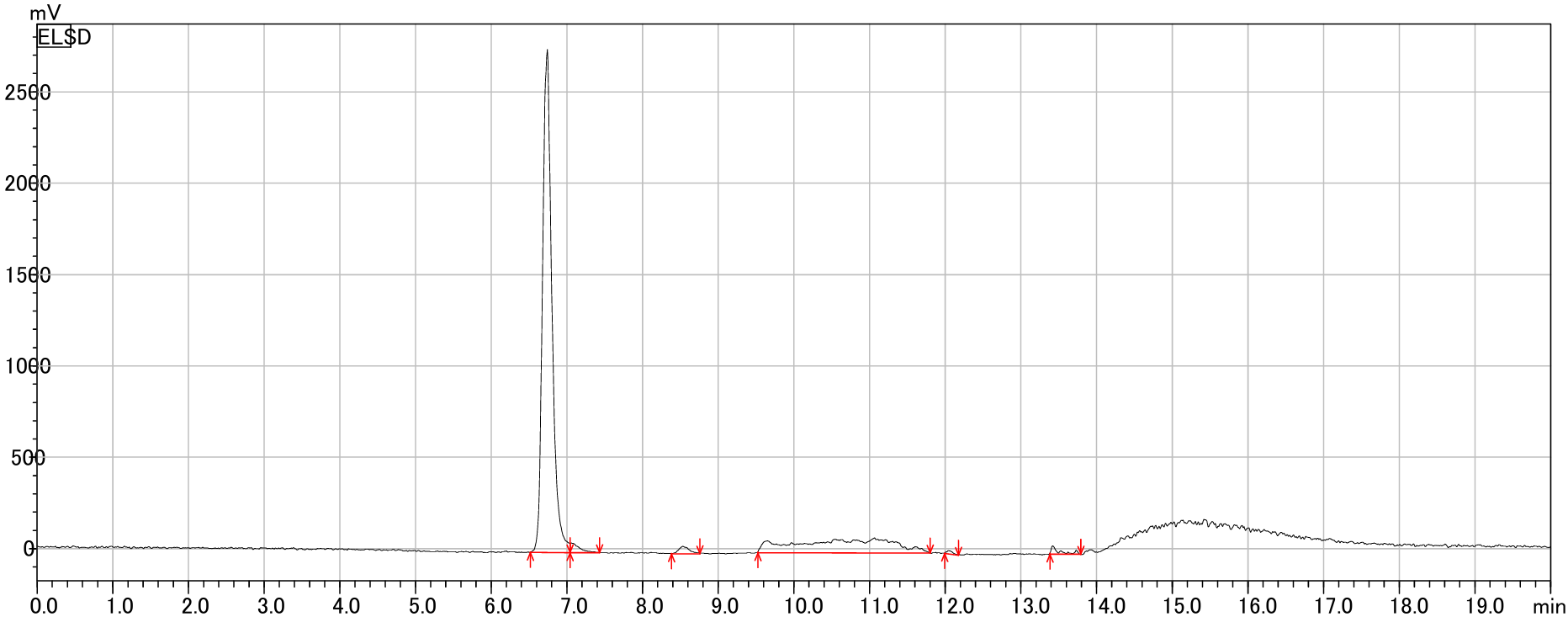


HPLC/ELSD: RT = 6.74 min, purity = 74.0%

1. **Synthesis of (R)-2,3-bis(oleoyloxy)propyl (2-((3-(heptan-4-yloxy)-3-oxopropyl)ammonio)ethyl) phosphate (DOPE-C4_α-3).**

4-Heptanol (116.2 mg, 1.0 mmol) and TEA (209 µL, 1.50 mmol) were dissolved in anhydrous DCM (4 mL) and then were added with acryloyl chloride (80.8 µL, 1.0 mmol) dropwise to an ice bath. The reaction mixture was stirred at ambient temperature for 2 hours. Solvent was removed *in vacuo*. The residue was suspended in hexane/AcOEt (9:1) and was passed through a silica pad. The filtrate was concentrated *in vacuo*. The crude product (heptan-4-yl acrylate) was used for the next reaction without further purification.

DOPE (598.5 mg, 0.80 mmol), the crude product (heptan-4-yl acrylate) (165 mg), and TEA (209 µL, 1.50 mol) were dissolved in chloroform (4 mL) and were stirred at 60 °C for 2 days under an Ar atmosphere. The reaction mixture was loaded onto a normal-phase column (Sfär Silica HC D, Biotage) and purified by flash chromatography with a gradient mobile phase of DCM and MeOH. This gave 445 mg (60.9%) of **DOPE-C4_α-3** as a yellow viscous oil.

^1^H NMR (500 MHz, CD_3_OD) σ: 0.88 (t, 12H), 1.21-1.38 (m, 40H), 1.50-1.63 (m, 8H), 2.01 (m, 8H), 2.31 (m, 4H), 2.80 (t, 2H), 3.33 (t, 4H), 3.96-4.18 (m, 7H), 4.42 (m, 1H), 4.95 (m, 1H), 5.22 (m, 1H), 5.33 (m, 4H).


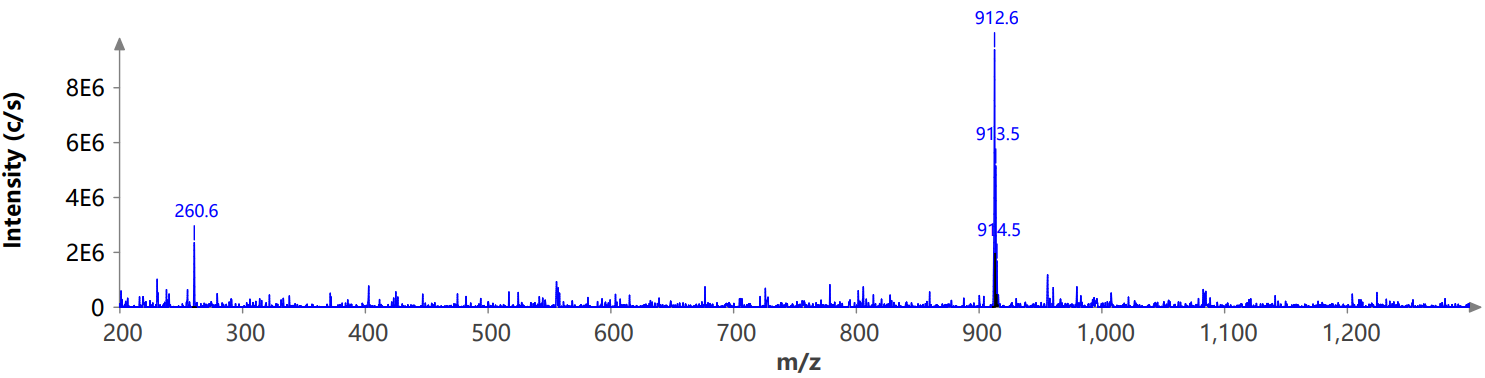


MS (ESI): m/z calculated for C_51_H_96_NO_10_P (M−H)^−^, 912.7; found, 912.6.

**
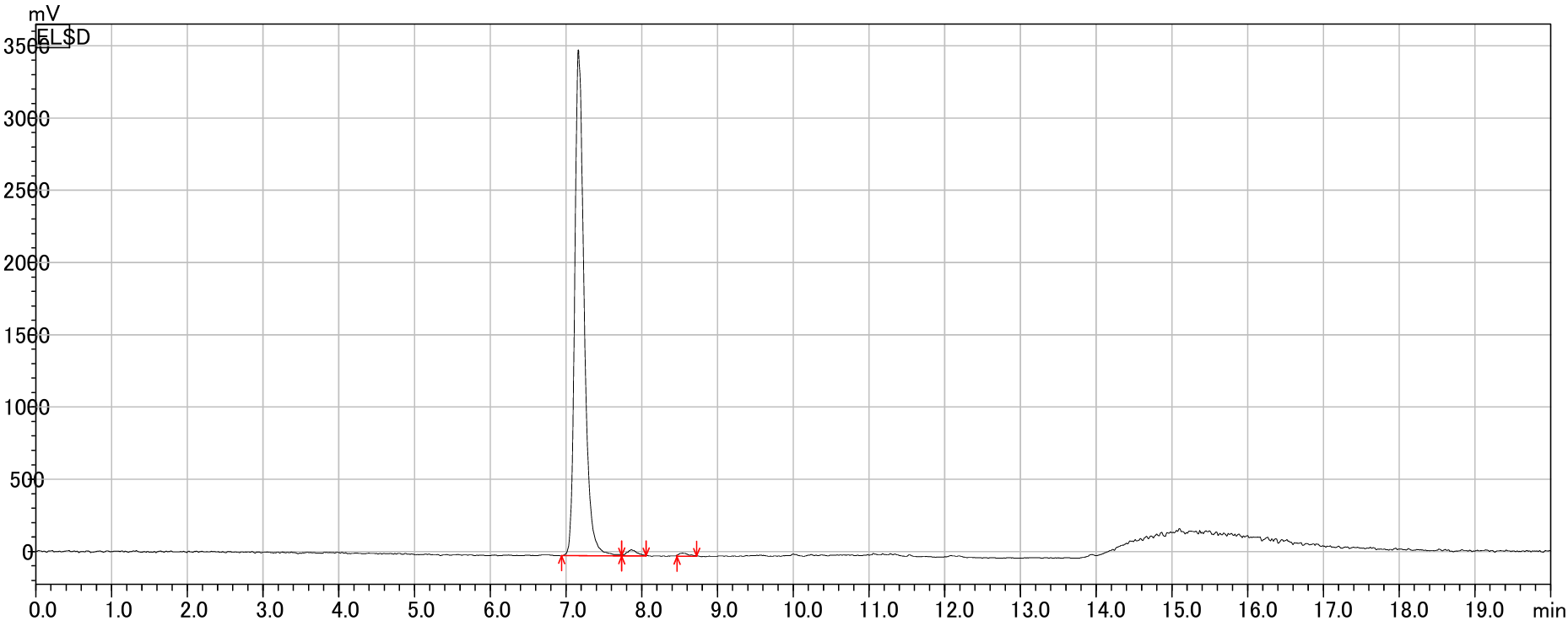
**

HPLC/ELSD: RT = 7.16 min, purity = 98.1%
